# Conceptualisation of financial capability in adults with acquired cognitive impairment: A qualitative evidence synthesis

**DOI:** 10.1177/02692155251347766

**Published:** 2025-06-12

**Authors:** Sarah Swan, Freyr Patterson, Terra M Bredy, Jennifer Fleming

**Affiliations:** School of Health and Rehabilitation Sciences, The University of Queensland, Brisbane, QLD, Australia

**Keywords:** Neurological rehabilitation, cognitive impairment, brain injury, dementia, activities of daily living

## Abstract

**Objective:**

To explore definitions, theoretical models and conceptual frameworks related to financial capability in adults with acquired cognitive impairment from acquired brain injury or other neurological disease, including dementia.

**Data sources:**

A systematic search of PubMed (inclusive of Medline), CINAHL, EMBASE, PsycINFO, ABI-inform, SCOPUS and the Cochrane database for papers published until May 2025.

**Review methods:**

A qualitative evidence synthesis approach was utilised in conjunction with the Preferred Reporting Items for Systematic Reviews and Meta-Analysis guidelines. Eligible papers articulated an original comprehensive definition and/or theoretical model or conceptual framework focused on financial capability in the target population. Papers were screened by two researchers, with methodological quality of included papers critically appraised. Data were extracted for tabulation and thematic synthesis, which was completed via coding and categorisation into descriptive and analytical themes.

**Results:**

The final analysis included 21 papers from the initial screening of 6516 papers. Fifteen discrete models were identified, with results indicating inconsistency in terminology use and meanings. Models that consider real-world performance within an individual's contextual environment are increasingly utilising economics related terminology such as financial capability. The majority of papers related to people with dementia and were multidisciplinary in authorship, or from the psychology literature. Themes found in the literature include the multi-dimensionality of financial capability, financial decision-making ability and exploitation risk for legal capacity, and the neuropathological cause of declining financial capability.

**Conclusion:**

Further research with the inclusion of the consumer lived experience is recommended to inform models of care for this complex area of practice.

## Introduction

Managing one's own financial matters is a critically important everyday life skill,^1^ and an ‘advanced’ activity of daily living that requires higher-order functional capacities.^2,3^ People who have acquired a cognitive impairment may experience difficulties with independent performance of financial tasks,^4–10^ and have increased vulnerability to financial exploitation.^11–14^ Acquired cognitive impairment may be the consequence of a stroke, brain tumour, traumatic brain injury, infection, poisoning, lack of oxygen; or other degenerative neurological disease or disability including organic dementias such as Alzheimer's disease that may lead to difficulties in physical, cognitive or emotional functioning.^15^

The health literature has been characterised by a lack of consistency in terminology use for defining a person's ability to manage financial affairs^16^; including the use of synonymy, with different terms being used to mean the same thing, and polysemy, where the same term is used with different meaning.^17^ In contrast, contemporary economics literature has championed the use of terminology such as ‘financial well-being’ and ‘financial capability’ in relation to the general population.^18–20^ Financial well-being has been defined in middle and high-income countries as ‘the extent to which someone is able to meet all their current commitments and needs comfortably and has the financial resilience to maintain this into the future’.^19^^(p.19)^ In this context, financial capability consists of ‘the behaviours and approaches to financial decision making that influence someone's financial well-being;’^20^^(p.14)^ or ‘how people put financial knowledge into practice’.^21^^(p.4)^ This review aims to clarify the conceptualisation of financial capability for people with acquired cognitive impairment due to brain injury or neurological disease. The research questions include: (1) How is financial capability defined in the literature? (2) What are the existing theoretical models or conceptual frameworks of financial capability? (3) How is financial capability conceptualised within those models/frameworks?

## Methods

### Protocol and registration

A qualitative evidence synthesis was conducted according to the Cochrane Effective Practice and Organisation of Care Group guidance for Qualitative Evidence Synthesis.^22^ A qualitative evidence synthesis is a type of qualitative systematic review with a discrete set of methodologies that synthesises the findings of multiple primary qualitative studies, with the aim of facilitating a greater depth of understanding in relation to a particular phenomenon of interest.^23,24^ It has been argued that when there is a paucity of primary qualitative studies related to an area of healthcare, that expert opinion can also be utilised to complement available evidence.^25^ Findings from primary qualitative studies and/or research papers that textually define or describe theoretical models or conceptual frameworks of financial capability were included in this review. Reporting follows the Preferred Reporting Items for Systematic Reviews and Meta-Analysis (PRISMA) guidelines.^26^ The synthesis protocol was registered at Open Science Framework (OSF Registries) and can be accessed at https://doi.org/10.17605/OSF.IO/J7USC.

### Search methods for identification of studies

The search strategy was developed in consultation with a health sciences librarian. The development of the research questions was supported by the use of the Sample, Phenomenon of Interest, Design, Evaluation, Research Type (SPIDER) tool^27^: *Sample:* Adults with acquired cognitive impairment (related to acquired brain injury or neurological disease); *Phenomenon of interest:* key concepts/definitions/theoretical models or conceptual frameworks related to financial capability; *Design:* N/A; *Evaluation:* N/A; *Research Type:* qualitative/descriptive.

Search terms were generated to include associated diagnostic terminology for acquired cognitive impairment and terminology linked to financial capability. A full systematic search using identified keywords and index terms was conducted across seven databases, including PubMed (inclusive of Medline), CINAHL, EMBASE, PsycINFO, ABI-inform, SCOPUS and the Cochrane database. The search strategy was adjusted to suit each database, with the full search strategy incorporating papers published from database inception to May 2025 (see Supplementary Materials for: Search strategy). The reference lists of final eligible papers were searched for relevant additional papers, alongside authors’ personal reference lists.

Search results were extracted to Endnote software and duplicates removed. Screening of papers was completed using Covidence systematic review software (Veritas Health Innovation, Melbourne Australia. Available at www.covidence.org). Two of three reviewers (SS & TB/FP) independently screened the titles and abstracts removing those not meeting the eligibility criteria, and any further duplicates. The full text of eligible papers, and papers where eligibility was not clear via title and abstract, were screened by two reviewers (SS & FP) to establish a final set of papers to include in the review. Disagreements between reviewers were resolved via discussion and consensus, with consultation with a third reviewer (JF) if required.

### Selection of studies

The synthesis included any literature that articulated an original comprehensive definition and/or theoretical model, or conceptual framework focused primarily on financial capability in adults with acquired cognitive impairment (including published studies, theses, and book chapters). For this study, a theoretical model or conceptual framework constituted ‘the specific perspective which a given researcher uses to explore, interpret or explain events or behaviour of the subjects or events s/he is studying.’^28^^(p.188)^ Inclusion criteria comprised papers related to adults (i.e. average age of participants is 18 years or over) with acquired cognitive impairment from diagnoses such as acquired brain injury, mild cognitive impairment or other degenerative neurological disease or disability including organic dementias such as Alzheimer's disease, multiple sclerosis, Parkinson's disease, etc. Included papers were related to any clinical practice setting, with full text available.

Papers that did not report a theoretical model or conceptual framework specific to financial capability, and/or an original comprehensive definition were excluded. Thereby, broader models or frameworks related to overall brain function or general cognitive function in which financial capability may have been described as a sub-component were not included. Papers that summarised previously published models/frameworks were not included unless the paper provided specific revisions or updates on an existing model/framework. Papers that related to paediatric populations, people with congenital intellectual disability and/or autistic spectrum disorder, or populations with discrete mental health conditions were excluded. Other exclusion criteria included papers not published in English where translation was not possible and conference or poster abstracts.

### Data extraction

Data extraction was completed by one author (SS) into a data extraction table. Data fields were based on Bacchi's^29^ ‘What's the problem represented to be?’ approach, which facilitates critical scrutiny of problem representation; and Mosey's^30^ extrapolation methods for the development, analysis and critique of theoretical conceptual practice models. Raw data extracted were: title of model/framework, author(s) and professional background, country of research origin, year of publication, target population for application, study design/purpose, definitions and key concepts, and model/framework theoretical foundation and overview.

### Assessing the methodological limitations of included studies

Methodological quality of included studies was measured with a six-item critical appraisal tool developed by Bergeron et al.^31^ for the systematic review of theories, models and frameworks; which was developed from guidance by Creswell^32^ and Caldwell et al.,^33^ and used in a recent qualitative systematic review.^34^ Three possible rating options are applied to each of the six questions: yes (1 point), no (0 points) or somewhat (0 points). Papers were categorised as being of strong (total quality score ≥4) or moderate quality (total quality score of ≤3). Papers with lower quality assessment scores were not excluded due to the limited literature pool; however, methodological limitations were used to assess our confidence in the review findings. Quality assessment was completed by one author (SS) and checked by all researchers.

### Data management, analysis and synthesis

Data management, analysis and synthesis were completed with Microsoft Excel. Synthesis of the qualitative data was flexibly approached, and drew on qualitative evidence synthesis techniques described by Flemming and Noyes.^23^ Theory classification type was assigned via the use of deductive coding, adapted from Sansonetti,^34^ with guidance from Lauffer's^35^ theory classification approach (see Table 1). The three-stage Thomas and Harden^36^ thematic synthesis approach was then utilised.

**Table 1.** Theory classification.

Theory classification	Definition	Example	Type of knowledge
Conceptual framework	‘A grouping of interrelated concepts used to explain a particular pattern or behaviour’	Engel et al.^37^ Proposes a dynamic framework from qualitative research that describes financial management activity processes.	Knowing ‘that’
Causal model	‘Abstraction of a cause-and-effect relationship’	Fenton et al.^38^ Model proposes that increased financial exploitation vulnerability is due to β-amyloid accumulation, and resulting structural and functional brain changes.	Knowing ‘that’
Working model	‘Preliminary formulation of a theory or program that is used as an initial guide for thought or action’	Moye and Marson.^39^ Provides preliminary commentary on contribution of declarative and procedural knowledge for financial judgement making ability.	Knowing ‘that’ and informing ‘how’
Programme model	‘A service or intervention approach’	Darzins et al.^40^ Proposes a six-step capacity assessment approach for managing property and finances.	Knowing ‘how’
Theory	‘Grouping of related facts, concepts, and hypotheses that is both descriptive and interpretive’	Nil identified in this review.	Knowing ‘that’

Source: Adapted with permission from Sansonetti,^34(p.8)^ according to Lauffer.^35(p.49)^

*Stage one:* First, line-by-line inductive coding of extracted data was conducted. Original objectives to extract and synthesise data separately in relation to financial capability definitions and conceptualisations were abandoned when it became apparent that definitions and their conceptualisation were intrinsically intertwined. Therefore, data regarding definitions and conceptualisation were extracted and synthesised collectively. The verbatim definitions and descriptions of concepts, models/frameworks of the included papers were entered into the spreadsheet. The text was then inductively coded, initially into free codes without hierarchical structure. As each study was coded, we added further data to the bank of codes and also added further codes when required. Utilising this approach allowed us to begin translating concepts between studies, essentially commencing the synthesis process.^36^ Prior to completion of this stage, consistency of interpretation was checked for all coded text, and to ensure that no further additional coding was required.^36^

*Stage two:* The next stage involved organising these ‘free codes’ into related ‘descriptive themes’. Reviewers commenced looking for similarities and differences between the codes to group them into hierarchically aligned descriptive themes.^36^ A draft summary of the findings across all included studies for the identified themes was then prepared by one author (SS) and discussed and agreed upon by all authors.

*Stage three:* The final stage involves ‘going beyond’ the findings of the descriptive themes from the primary studies to develop analytical themes, and is the most controversial stage of qualitative synthesis due to dependence on reviewer judgment and insights.^36^ This stage was completed independently by reviewers (SS, FP and& JF) and then collectively. Via cyclical discussion and debate, a number of analytical themes were found to provide sufficient explanation of the initial descriptive themes.

### Assessing confidence in the review findings

The GRADE-CERQual (Confidence in the Evidence from Reviews of Qualitative Research) approach was used to assess confidence in each finding.^41^ GRADE-CERQual assesses confidence in the evidence, based on four key components: (1) methodological limitations of included studies; (2) coherence of the review finding; (3) adequacy of the data contributing to a review finding; (4) relevance of the included studies to the review question. Initial GRADE-CERQual ratings were completed by the first author (SS) and checked with other authors. A judgement was made about the overall confidence (high, moderate, low, or very low) in the evidence supporting the review findings after the assessment of each of the four components was completed.

## Results

### Results of the search

**Figure 1.**
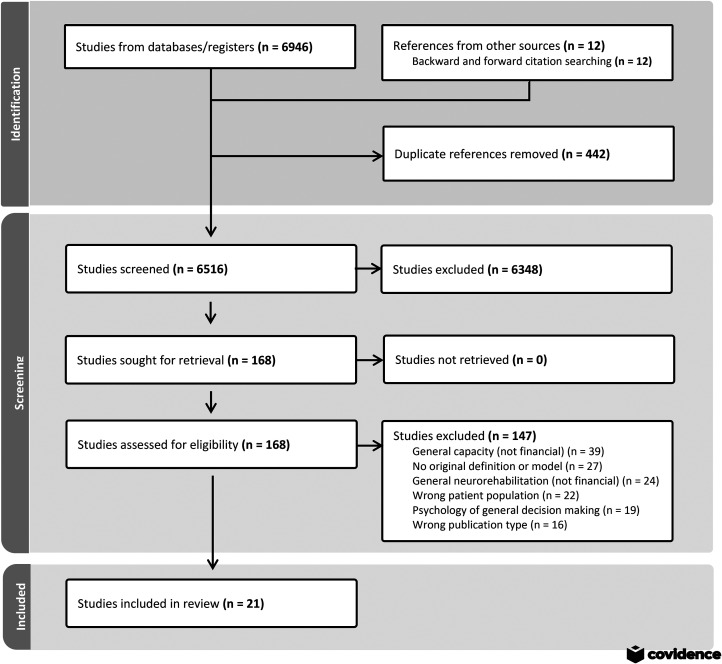
PRISMA flow diagram.

A total of 6946 papers were located from a systematic search of the seven databases. See Figure 1 for the PRISMA flow diagram of papers identified and selected for the review. Following duplicate removal and the addition of papers from other sources including reference lists, 6516 papers were screened by title and abstract, with full-text review resulting in 21 eligible papers. Where multiple papers presented the same model, the review included the paper in which the model was originally proposed and any further papers in which the model was altered or expanded upon. A total of 15 discrete models were identified across 21 papers. Some of the 21 included papers presented models at progressive stages of development, and one paper presented expansions of two previously published models.^1^

### Description of the studies

Table 2 presents a descriptive summary of the 15 identified models and frameworks as they were published chronologically. An extended version of Table 2 is also available within Supplementary Materials that offers further description of models and definitions of key terms within papers. Papers were published from 2000 to 2024, and included 18 journal articles, 2 books and 1 dissertation. Authors came from the United States (*n* = 12), Canada (*n* = 4), Australia (*n* = 4), and the United Kingdom (*n* = 1). Professional backgrounds of authors were varied, with some papers being authored by singular professional backgrounds, including psychology/neuropsychology (*n* = 6) and geriatric medicine.^40^ However, the majority were multidisciplinary (*n* = 13) in nature, with a recent paper by a psychology team also including authors with lived experience as carers of people with dementia.^42^ Target population varied, with papers relating to people with mild cognitive impairment/Alzheimer's disease (*n* = 8), adults with acquired cognitive impairment (*n* = 3), acquired brain injury (*n* = 3), and those with ‘questionable capacity’ (*n* = 1). Theory classification indicated papers included conceptual frameworks (*n* = 3), programme models (*n* = 7), causal models (*n* = 2), and working models (*n* = 8). One paper presented a conceptual framework and programme model.^13^ Another presented a causal model, conceptual framework and programme model.^43^ Henceforth, all such theory classifications will be referred to as a ‘model’.

**Table 2.** Descriptive information for included papers, models and conceptual frameworks.

	Author(s) professional background	Target population	Theory classification	Author (year), country of origin, type of publication	Study design/purpose	Key terms (descriptions in Supplementary Materials)	Framework/model overview	Quality assess-ment rating
1. Who can decide? Property and finance	Geriatric medicine	People with ‘question-able capacity’	Programme model	Darzins, Molloy, Strang et al.^40^ Australia, Book.	Outline of a six-step capacity assessment process for property /finance.	Capacity (for property)	Proposes six steps in assessing capacity to manage property including: (1) valid trigger; (2) engage the person in the assessment process; (3) gather facts to base assessment on including person's situation, choices available and foreseeable consequences; (4) educate person; (5) capacity assessment via structured interview (“decisional aid for capacity to manage property” provided); (6) act on assessment results.	4/6 Strong
2. Conceptual model of financial capacity	Neuropsychology (one author with dual legal qualification)/Medicine	Alzheimer's disease/Dementia	Programme model	Marson, Sawrie, Snyder, et al.^2^ United States. Journal article.	Development of a conceptual model and prototype instrument to investigate financial capacity in people with Alzheimer's disease.	Financial capacity	Model has two levels: (1) General domains of financial activity; (2) Specific financial abilities or tasks that operationalise individual domains. Includes 14 specific financial tasks within six domains: basic monetary skills, financial conceptual knowledge, cash transactions, checkbook management, bank statement management and financial judgement. Forms basis of the financial capacity instrument (FCI).	2/6 Moderate
				Model revision 1: Earnst, Wadley, Aldridge, et al.^44^ United States, Journal article.	Revised model approved by an expert panel and used to examine relationship between working memory and financial abilities in people with Alzheimer's disease.	Financial capacity	Revised model has three levels: (1) specific financial abilities (tasks); (2) broader financial activities (domains); (3) overall financial capacity (two global scores). Includes 19 financial tasks within 8 domains. Two new domains include bill payment and assets and estate arrangements. Addition of ‘core knowledge type’ to tasks (declarative/ procedural/judgemental). Basis of revised FCI.	2/6 Moderate
				Model revision 2: Griffith, Belue, Sicola, et al.^3^ United States, Journal article.	Revised model presented in the context of use for assessment of financial capacity in people with mild cognitive impairment.	Financial capacity	Revised model has three levels as previously, and two global scores. Includes 18 financial tasks within nine domains. Additional domain is Investment decision-making. Addition of ‘difficulty’ classification to tasks (simple/complex). Basis of revised FCI.	2/6 Moderate
				Model reframed as: *Clinical model: Financial capacity as financial skills relevant to independence* Marson^1^ United States, Journal article.	Overview of conceptual models of financial capacity, and guidance of assessment processes.	Financial capacity	Model essentially unchanged from 2003 revision, however titled differently to reflect an extension of early IADL assessment proposed by Lawton and Brody^45^ that considers financial skills and judgements as relevant to independent living.	3/6 Moderate
3. Financial Competency Model	Psychology	Adults with cognitive impairment	Working model	Webber, Reeve, Kershaw, et al.^46^ Australia, Journal article.	Early development of model briefly described based on literature review and survey of professionals (*n* = 64).	Financial competence	Brief description of model based on 63 fiscal skills derived from literature review and rated by professionals. Factor analysis indicated four dimensions of importance: financial judgement, everyday financial abilities, estate management, debt management.	1/6 Moderate
			Conceptual framework	Model revised: Kershaw and Webber^47^ Australia, Journal article.	Further model development based on factor analysis of a wider survey of professionals (*n* = 212) about the importance of financial tasks and skills for financial competence applicable to a scenario of an adult with cognitive impairment.	Financial competence	Four dimensions identified by Webber et al.^46^ supported by larger survey, with a further two dimensions identified. Consists of six dimensions that emerged from factor analysis of 61 original items including: everyday financial abilities, financial judgement, estate management, debt management, cognitive ability (basic literacy and numeracy skills) and support resources (assistance provided by others and the individual's assistance-seeking skills).	4/6 Strong
			Programme model	Model evaluation: Kershaw and Webber^48^ Australia, Journal article.	Development and validation of the Financial Competence Assessment Inventory (FCAI) based on the financial competency model.	Financial competence	FCAI has a structured interview format with 41 questions/tasks related to financial abilities. Results provided supporting evidence for the six-dimension financial competency model measured by the FCAI. FCAI items were also recoded and used to evaluate the Appelbaum and Grisso^49^ four legal criteria ‘understanding’, ‘appreciation’, ‘reasoning’, and ‘‘expressing a choice’’.	4/6 Strong
4. Working model of financial capacity	Clinical psychology/ Neuropsychology (dual Legal qualification)	Older adults, including those with Alzheimer's disease.	Working model	Moye and Marson^39^ United States, Journal article.	Overview of existing research related to medical decision-making capacity and financial capacity.	Financial capacity	Model combines clinical and cognitive neuropsychological aspects, highlighting the contribution of a person's declarative knowledge, procedural knowledge and judgement abilities. (1) Declarative knowledge includes a person's ability to outline details regarding personal finances, and general financial facts (e.g. value of currency). (2) Procedural knowledge is related to learned motor-based financial skills (e.g. complete simple online banking tasks); (3) Judgement-based abilities, which include the utilisation of declarative and procedural financial knowledge and skills to make financial judgements.	2/6 Moderate
				Model reframed as: *Cognitive Psychological Model: Financial capacity as types of financial knowledge.* Marson^1^ United States, Journal article.	Overview of conceptual models of financial capacity, and guidance of assessment processes.	Financial capacity	Model essentially unchanged from original publication, however titled differently to acknowledge cognitive psychological basis. Further elaboration provided on processes and relationships involved in declarative knowledge, procedural knowledge and judgement.	3/6 Moderate
5. Conceptual Model of Declining Financial Capacity in amnestic mild cognitive impairment (MCI) and Alzheimer's disease	Neuropsychology	Alzheimer's disease and amnestic mild cognitive impairment	Causal model	Copeland^50^ United States, PhD Dissertation. Supervisors: Triebel and Marson.	Doctoral dissertation with model linked to imaging study that had ‘somewhat consistent’ results to hypotheses.	Financial capacity	Neuropathological changes that occur in the brain in people with amnestic mild cognitive impairment and Alzheimer's disease (cortical thinning of the grey mantle in specific regions of interest) underlie specific neurocognitive impairment in arithmetic (inferior parietal cortex), attention and executive function (precuneus/superior frontal cortex) and memory and learning (parahippocampal gyrus/entorhinal cortex) that contribute to a decline in everyday financial skills.	6/6 Strong
6. Financial Decisional Abilities Model	Clinical Psychology/Neuropsychology/Clinical Geropsychology/Gerontology	Older adults, including those with cognitive impairment/dementia.	Conceptual framework/Programme model	Lichtenberg, Stoltman, Thicker, et al.^13^ United States, Journal article.	Literature review derived proposed conceptual model with focus on general decisional abilities and financial exploitation. Model refined by two groups of experts (*n* = 6) and (*n* = 12), with assessment instrument developed based on model.	Financial decisional abilities, financial exploitation.	Model encompasses contextual factors and intellectual factors, and whether there is consistency with a person's values. Contextual factors include financial situational awareness, past financial exploitation, psychological vulnerability, and undue influence. Intellectual factors encompass functional abilities to make a choice, explain rationale, understand, and appreciated relevant factors involved. Basis of the Lichtenberg financial decision rating scale (LFDRS).	6/6 Strong
			Programme model	Later referred to as: *Financial Decisional Capacity Model* Lichtenberg, Ocepek-Welikson, Ficker, et al.^51^ United States, Journal article.	Examined empirical support for conceptual model, and psychometric properties of the related measurement scale, the Lichtenberg Financial Decision Rating Scale (LFDRS).	Financial decision making, financial exploitation.	Model re-titled and study results confirmed the reliability of the LFDRS and supported the conceptual model. LFDRS can be utilised for clinical assessment of real-world financial decision-making, and is unique in focusing on actual financial decisions with consideration of contextual variables.	6/6 Strong
7. Social Cognitive Neuroscience Model for Assessing Financial Exploitation Risk	Psychology/Neuroscience/Neurology	Older adulthood, mild cognitive impairment and dementia.	Causal model/Conceptual framework/Programme model	Spreng, Karlawish and Marson^43^ United States, Journal article.	Literature review derived novel framework and assessment tool of financial exploitation risk in older adulthood following age-related changes in the brain, cognition, and social functioning.	Financial exploitation	Two neurally and behaviourally distinct interacting pathways (cognitive capacity and social capacity) are associated with structural and functional brain changes that may increase financial exploitation risk. Change in cognitive capacity is associated with possible increase in exploitation due to a decline in financial skills (decline in fluid reasoning ability due to changes in lateral frontal and parietal brain regions). Changes in social cognition increase vulnerability to financial exploitation via social influence, deception, or coercion (decline in social capacity due to changes in default network brain regions, or their interactions with affect processing subcortical brain regions). Basis of the Financial Competence in Everyday Decision-making (FCED) assessment tool.	6/6 Strong
8. Institute of Medicine Conceptual Model of Financial Capability	Psychiatry/Neurology/Medicine/Public Health/Social Work/Neuropsychology/Occupational Therapy	Adults with disabilities (including cognitive impairment)	Conceptual framework	National Academies of Sciences, Engineering and Medicine^52^ United States, Book.	Conceptual model proposed within an evaluation of the Social Security Administration's (SSA's) capability determination processes for adult beneficiaries in the United States. Derived from literature review, review of other capability determination processes, and multidisciplinary expert consensus.	Financial capability, Financial performance, Financial competence, Financial judgement, Financial knowledge.	Promotes clear definitions of concepts related to financial capability and their relationships. Makes the distinction between financial competence and financial performance. While financial skills, knowledge and judgement contribute to financial competence; financial performance also includes additional contextual factors such as stresses, supports, cues or resources in a person's actual environment. Acknowledges that a person may be financially competent in a controlled environment, but that this may not extend to a real-world environment; or that conversely someone who is not financially competent in a controlled environment may demonstrate appropriate financial performance with support systems or assistance in place, therefore being financially capable in a real-world environment.	6/6 Strong
9. Concept of financial management	Occupational Therapy/Neuropsychology	Acquired cognitive impairment	Working model	Engel, Bar, Beaton, et al.^53^ Canada, Journal article.	Systematic review that outlines the concept of financial management and identifies instruments that quantify financial management skills in adults with acquired cognitive impairment.	Financial management skills	Financial management skills outlined as encompassing multiple levels of functioning in relation to the International Classification of Functioning, Disability and Health (body functions/mental functions, activity and participation). Distinction is made between financial management skills and the legal construct of financial capacity/competency, which is decided in a court of law. Financial management skills assessment promoted as a component of capacity/competency assessment.	3/6 Moderate
10. Four-factor structure of financial capacity	Neuropsychology (one author with dual Legal qualification)/Medicine	Older adulthood, mild cognitive impairment and dementia.	Working model	Gerstenecker, Triebel, Eakin, et al.^54^ United States, Journal article.	Proposed model based on exploration of the factor structure of financial capacity by using a performance-based assessment (Financial Capacity Instrument) as a proxy for the construct.	Financial capacity	Four extracted factors identified to be core components and accounted for 46% of the variance. The four factors included: (1) Basic monetary knowledge and calculation skills, including semantic knowledge of currency values and arithmetic calculation skills (35.8% of shared variance); (2) Financial judgement, comprising items targeted at ability to detect and avoid fraudulent ‘scams’ (5.2% of shared variance); *(*3) Financial conceptual knowledge, consisting of items ascertaining comprehension of simple financial concepts such as knowing what a debt is and reasons for banking money etc. (2.6% of shared variance); *(*4) Financial procedural knowledge, related to ability to write a cheque/record a transaction in a cheque register (2.6% of shared variance).	5/6 Strong
11. The Financial Management Activity Process (FMAP)	Occupational Therapy/Neuropsychology	Acquired brain injury	Conceptual framework	Engel, Beaton, Green, et al.^37^ Canada, Journal article.	Conceptual framework to guide ABI rehabilitation derived from grounded theory qualitative study regarding the experiences, actions and processes of adults living with ABI and close others who assist them with financial management tasks.	Financial management	Framework has three key phases that are fluid, with dynamic interrelated interactions: (1) Identifying a financial management activity that requires completion; (2) Exploration of ‘filter factors’ related to the person, environment or activity that may influence task completion; (3) Personalising process, whereby trusted strategies and processes are used to facilitate task completion including simplifying and organising; recording and checking records; scheduling; setting personalised financial management policies; using or directing assistance; and obtaining advice or knowledge. Strategies and processes are applied within a ‘lens of trust’, for themselves, others, technology, and organisations. Technology options identified to assist in strategy application include low (pencil/paper) and high technology (smartphones, internet etc.), allowing the framework to remain relevant as technology advances.	6/6 Strong
12. Financial exploitation vulnerability manifests as an early behavioural sign of underlying Alzheimer's disease related neuro-pathology	Psychology/Medicine/Gerontology	Adults at risk/or who have Alzheimer's dementia	Causal model	Fenton, Weissberger, Boyle, et al.^38^ United States. Journal article.	Literature review derived model based on review of neuroimaging, neuropathological, and cognitive correlates identified as underlying financial exploitation vulnerability in individuals at risk of cognitive decline.	Financial exploitation	Proposes that early accumulation of Alzheimer's disease related neuropathology (β-amyloid within the default mode network [DMN]) and resulting structural/functional brain changes (neural connectivity disruption between brain regions involved in decision-making, risk assessment, value judgements) manifest in increased financial exploitation vulnerability that is seen in subtle changes in cognition and impaired decision-making.	3/6 Moderate
13. The digitisation of financial management skills in dementia since Covid-19	Psychology/Carers of people with dementia	Dementia	Working model	Giebel, Halpin, Tottie, et al.^42^ United Kingdom, Journal article.	A qualitative interview study exploring the effect of the COVID-19 pandemic and digitalisation of financial management skills in people with dementia and their carers.	Financial management skills	Five overarching themes (with subthemes) found, including: (1) Potential dangers of early loss of finance management skills; (2) Face-to-face shopping experiences and skills (lack of support and recognition from staff and other shoppers, feeling anxious and rushed, maintaining independence or supporting someone to be); (3) Barriers and facilitators of moving to digital (online use benefits/disadvantages); (4) COVID-19 triggered fast-tracked digitisation; (5) Carer impact due to supporting someone with finances.	5/6 Strong
14. Contextual factors of financial capability	Occupational Therapy/Neuropsychology/Nursing/Psychology/Public Health/Social work	Acquired brain injury	Working model	Engel, Arowolo, Ewesesan, et al.^55^ Canada, Journal article.	A qualitative photovoice study to identify financial capability and financial well-being contextual factors (barriers and facilitators) following acquired brain injury.	Financial capability, Financial well-being.	Four themes of contextual factors generated including: (1) Economic context (finding adequate financial resources/income, making sense of complex financial information and process); (2) Social context (having a trusted person to provide assistance/guidance, bias and stigma related to ‘invisible’ disability); (3) Physical and sensory environment (layout and design of financial institutions); (4) Technology environment (benefits and challenges).	5/6 Strong
15. Financial capability and financial well-being of adults with ABI	Occupational Therapy/Computer science/Nursing/Psychology/Public Health/Social work	Acquired brain injury	Working model	Engel, Ewesesan, Arowolo, et al.^56^ Canada, Journal article.	A pilot cross-sectional survey of adults with acquired brain injury and close others to examine financial capability and financial well-being experiences and challenges.	Financial capability, Financial well-being.	Four categories were developed from coding of narrative responses including: (1) Complexity of financial capability (including lack of support services); (2) Accessing and navigating financial well-being resources (availability, eligibility and access issues); (3) Increased financial exploitation vulnerability (perceived risk of abuse or fraud); (4) Implications on financial well-being from downward shift in income and employment.	5/6 Strong

### Methodological limitations of the studies

A strong quality rating was given to 13 papers, with 8 papers rated as moderate quality (see Table 2 and Supplementary Materials for: Methodological quality assessment). A strength of most of the included papers was that the theoretical frameworks utilised to guide the study were easily linked with the problem of financial capability. However, while just over half of the papers distinctly identified and described the perspective or theoretical lens used to guide the study, most of the papers rated as moderate quality lacked clear statements and perspectives in relation to this criterion. The majority of papers with strong quality ratings were superior in the identification and justification of the methodology for model development. A strength of most papers was the provision of adequate definitions and descriptions of model and framework concepts. Moderately rated papers generally had limitations in the identification of the relationships amongst the concepts presented. This tended to particularly apply to papers that presented programme models with a service or assessment approach whereby concepts were more likely to be presented individually, with a lack of description of the interaction between concepts. While some of the primary qualitative studies outlined conceptual themes, further clarity of concept interplay would have raised quality ratings.

### Review findings

The first stage of inductive coding created an initial bank of 44 codes. The second stage resulted in seven descriptive themes that the codes were hierarchically aligned to. The third stage resulted in three analytical themes, with accompanying sub-themes which are described below (see Supplementary Materials for: Coding matrix).

#### Theme 1: Multi-dimensionality of financial capability

This theme focused on how financial capability can be considered a multi-dimensional construct, with subthemes including the multi-dimensionality of financial capability tasks; the multi-dimensionality of financial knowledge, skills, and abilities; real-world processes and performance; and the extent of individualisation. Definitions that reflected this theme related to financial capacity, financial competence, financial management, financial performance, financial capability and financial well-being. Full definitions from individual papers are provided in Supplementary Materials (see: Extended version Table 2) and outlined further where pertinent within the sub-theme results.

*Sub-theme 1: Multi-dimensionality of financial capability tasks.* Financial capability is viewed or acknowledged within five models as a multi-dimensional construct consisting of several domains or dimensions in respect to financial tasks relevant to maintaining financial independence.^1–3,39,44,46–48,53,54^ Some of these models, such as ‘the conceptual model of financial capacity’ proposed by Marson's research group^1–3,44^ and the ‘financial competency model’ by Kershaw and Webber,^47,48,54^ include tasks such as basic monetary management, cash/cheque transactions, bill payments, bank statement management, asset/estate management, and financial conceptual knowledge and judgement. Marson et al.^2^^(p.878)^ used the term ‘financial capacity’ and conceptualise it as ‘a series of discrete, clinically relevant domains of activity rather than as a unitary construct’. Due to the view that legal definitions focus more on ‘incapacity’, Kershaw and Webber^47^^(pp.338–339)^ preferred to use the term ‘competence’ to describe ‘the ability to adequately carry out specific tasks that related to a domain’.

Four models consider financial skills, abilities, and task performance within a structure of discrete financial domains or dimensions.^1–3,39,44,46–48,54^ Two of these are programme models related to assessment instruments: the Financial Capacity Instrument (FCI) based on ‘the conceptual model of financial capacity’,^1–3,44^ and the Financial Competence Assessment Inventory (FCAI)^48^ generated from the ‘financial competency model’.^47^ Based on an initial description by Marson and colleagues,^2^ financial capacity is recognised explicitly within three additional models as an advanced instrumental activity of daily living, being distinct from other household or personal care activities of daily living.^39,42,50^ Similarly, other models view financial management or financial capability as a vital ‘occupation’ requiring appropriate performance of tasks for independent community living and quality of life.^37,53,55,56^

*Sub-theme 2: Multidimensionality of financial knowledge, skills and abilities.* Financial knowledge, skills and abilities are acknowledged as underpinning financial capability and performance of financial tasks in all 15 models. A distinction is made between the contribution of cognitive and judgement skills versus functional or performance skills to the completion of financial tasks in nine models.^1–3,13,37,39,40,42,44,46–48,51,52,54^ In some models this division is clearly evident with separate knowledge and performance domains relevant to specific tasks as discussed in the first subtheme.^1–3,44,47,48,54^ Other models more subtlety recognise this distinction, with some acknowledging that proficient cognitive and judgement skills do not necessarily lead to independent performance of financial tasks due to a variety of task demands or social, environmental, or contextual factors; or alternatively that independent performance of financial tasks is not always necessarily tied to capable cognition or financial judgment.^13,37,40,42,51,52^ This is illustrated in qualitative research findings. For example, a participant with acquired brain injury who had sound financial knowledge disclosed that discussions with a financial advisor were compromised by forgetfulness.^37^ Whereas, a person with dementia who had declining financial knowledge and ability was still able to perform financial tasks such as paying for an item with a bank card that a carer had imposed spending limits on.^42^

General knowledge of assets, debts and everyday use of money is discussed in five models.^1–3,39,40,44,47,48,54^ Two of these models consider this knowledge in relation to a person's individual situation^1,39,40^; whereas the other three refer to these items more as general concepts, or present primarily hypothetical situations within a programme model.^1–3,44,47,48,54^ Four of the models offer a more conceptual overview of financial capability components, including knowledge, skills and abilities.^39,52–54^ One of these is a working model of financial capacity by Moye and Marson,^1,39^ which proposes that making financial judgements is dependent on the use of both declarative knowledge and procedural knowledge. Declarative knowledge includes a person's ability to outline details regarding personal finances, and general financial facts such as the value of currency and meaning of terms such as loan and debt.^1,39^ Procedural knowledge is related to learned motor-based financial skills including how to use an automatic teller machine, count change, write cheques or complete simple online banking tasks.^1,39^ A similar comprehensive conceptualisation, including declarative and procedural components of financial knowledge, is found in the ‘Institute of Medicine conceptual model of financial capability’.^52^ This model was developed in the United States by an expert multidisciplinary committee formed to evaluate the capability determination process for adults with cognitive impairment to receive government benefits.^52^ It views ‘financial competence’ as being underpinned by financial knowledge and judgement, demonstrated by financial skills that are typically assessed within a controlled (e.g. clinical or office) setting.^52^

*Sub-theme 3: Real-world processes and performance.* Three models explicitly acknowledge that financial knowledge, judgement, and skills are not always indicative of real-world performance of financial tasks.^37,42,52^ Two of these models are from qualitative research with people with dementia^42^ and acquired brain injury^37^ and their carers, with practical examples provided in the previous subtheme. The third model, the ‘Institute of Medicine conceptual model of financial capability’ recognises that a person may be financially competent in a controlled setting, but that this may not extend to a real-world environment; or conversely that someone who is not financially competent in a controlled setting may demonstrate appropriate financial performance with support systems or assistance in place in a real-world environment.^52^ The model regards an individual's success in managing financial demands within their own contextual environment including supports, resources, stresses and cues as a person's ‘financial performance’.^52^ The model proposes that ‘financial capability’ or ‘the management or direction of the management of one's funds in a way that routinely meets one's best interests’^52^^(p.5)^ is shaped by both financial competence and financial performance.

Consideration of ‘how’ financial tasks are carried out, including processes and actions in a real-world environment, is considered in five models.^37,40,42,52,55^ The ‘financial management activity process’ (FMAP) by Engel and colleagues,^37^ is a comprehensive, fluid and dynamic conceptual framework derived from qualitative research to guide acquired brain injury rehabilitation. The framework proposes three key phases, including a person identifying a financial management activity that requires completion; exploration of ‘filter factors’ related to the person, environment or activity that may influence task completion; and a personalising process, whereby strategies and processes are applied within a ‘lens of trust’ to facilitate task completion.^37^ A variety of high and low technology options are identified to assist in strategy application, arguably allowing the framework to remain relevant within the context of technological advances.^37^

Consideration of financial capability in relation to the International Classification of Functioning, Disability and Health (ICF) is discussed explicitly in relation to two of the models.^52,53^ The ICF recognises the relevance of a person's real-life environmental context in addition to other factors relating to health conditions such as body functions and structures and activity participation.^52,53^ Three recent models based on qualitative research consider how the complexity of financial processes and information could be a potential participation barrier for completion of real-world financial tasks for people with acquired brain injury^55,56^ and dementia.^42^ Availability, or a lack of support provided by external statutory bodies, banks or financial institutions to people with acquired cognitive impairment is considered in five of the models.^37,42,52,55,56^ Two models related to people with acquired brain injury^55^ and dementia^42^ suggest that bias and stigma from ‘invisible disability’ can lead to a lack of support or recognition of need for further information, time or support to complete financial tasks. One model considers the potential social benefits to people with dementia of completing financial management tasks in-person, such as shopping;^42^ whereas two models consider the potential barriers or facilitators relating to the layout and design of physical environments where financial management tasks take place for people with acquired brain injury.^37,55^

The changing technological financial landscape was discussed in relation to three models, with consideration of the use of both low and high technology for strategies to aid in financial task participation.^37,42,55^ These models also consider the qualitatively reported benefits and the disadvantages of the ongoing digitisation of finances. Reported benefits include the use of carer-monitored debit cards sustaining longer periods of participation in financial tasks, direct debiting bills, and greater independence with online banking and purchases for people who were previously adept with technology use.^37,42,55^ Disadvantages include potential difficulty learning or sustaining use of technology and increased support needs to monitor spending, set limits or complete tasks for people who have difficulty with online banking or shopping.^37,42,55^

*Sub-theme 4: Extent of individualisation.* The majority of models describe consideration of a person's individual context and/or contextual factors as important financial capability factors; however, not all models provide practical guidance on how this can be achieved. Five models consider gathering information about a person's individual financial context.^13,37,39,40,47,48,51^ Two of these models are the ‘cognitive psychological model’ where a person's awareness of their own financial situation is their declarative knowledge^1,39^; and the FMAP conceptual framework where a person needs to have knowledge of their own situation to identify financial tasks that require completion.^37^ Three programme models aimed at assessing financial capability provide specific direction to explore these individual contextual aspects.^40,48,51^ Firstly, Darzins and colleagues’ programme model^40^ proposes a six-step process to assess overall capacity to manage property and finance that is centred on an individual's situation, which includes gathering information about a person's financial situation, providing education regarding choices and foreseeable consequences, and assessing ability via a structured interview. Secondly, the ‘financial competency model’ and resulting FCAI assessment includes a few questions regarding source of income and level of independence with management of finances and paying bills.^57^ Instruction on how to ascertain the correctness of this information is not provided in the formal assessment guide, therefore, there is an underlying assumption of assessor knowledge.^57^ Thirdly, Lichtenberg et al.'s model and resulting assessment^51^ provide more flexible guidance on the assessment of an individual's ability to make specific sentinel financial decisions within their own personal context.

The importance of independent financial capability for autonomous community living is promoted by six models,^1–3,37,39,40,44,50,54^ while three models discuss promotion of a person's own financial well-being.^1–3,39,44,54^ Three models have an explicit focus on ‘person-centredness’.^13,37,43,51^ Two recent models based on qualitative research with people with acquired brain injury consider available financial resources within an employment and economic context.^55,56^ Consideration of a person directing the management of their finances with assistance or support, rather than complete independence or reliance on a substitute decision-maker, was discussed by five models.^37,42,52,55,56^ Four of these models discuss assistance or personalised strategies potentially required by people with ABI^37,55,56^ or dementia^42^ to complete financial tasks. The perspectives of significant others were explicitly considered in relation to five models^37,40,42,52,56^; but consideration of the impact on carers in relation to providing assistance, and a person's acceptance of that assistance, was only considered by one model.^42^

More recent terminology reflects the broadening conceptualisation of financial capability to include consideration of an individual's contextual factors. Engel and colleagues define ‘financial capability’ as ‘the knowledge, skills, attitudes/confidence, and applied behaviours related to managing money, accessing financial resources, planning and making choices related to finances, and securing financial-related help when needed’.^56^^(p.2)^ Financial capability arguably contributes to a person's overall ‘financial well-being’, which encompasses a person's financial outcomes, dependent on the ability to meet current and future financial needs within subjectively felt financial stress.^55^

#### Theme 2: Financial decision-making ability and exploitation risk for legal capacity

This theme focused on the ability to make financial decisions in respect to a person's legal capacity, alongside the risk for financial exploitation when a person's ability to make decisions is in decline. This theme included two sub-themes with the first related to legal capacity, focused on competence, financial judgement and financial decision-making, and the second on financial exploitation.

*Sub-theme 1: Legal capacity.* The concept of ‘legal competency’ is acknowledged in models by Marson's American research group^1,2^ and early work by Australians Webber et al.^46^ This is largely based on original work regarding treatment consent capacity from the United States by Appelbaum and Grisso,^49^ who define ‘competence’ as a legal concept, determined only via formal legal proceedings that examine a person's decision-making capacities. Alternatively, according to Darzins et al.^40^ from Australia, ‘capacity’ and ‘lack of capacity’ are both legal concepts, with the definition of capacity being determined by the legal definition in each particular jurisdiction. Darzins et al.^40^ argued that an individual's understanding and appreciation of information determines capacity, rather than task performance ability. Therefore, healthcare workers are positioned to provide expert testimony to courts rather than determine capacity itself.^40^ Later models by Kershaw and Webber^47,48^ in Australia and Lichtenberg et al.^51^ in the United States also adopted the terminology of ‘capacity’ to refer to a legal framework.

Four models specifically considered criteria for legal capacity,^13,40,47,48,51,52^ citing widely acknowledged legal principles, including the ability to express understanding, appreciation, reasoning and choice.^49^ The ‘Institute of Medicine conceptual model of financial capability’ encompasses these principles in its definition of ‘financial judgement’, as: ‘possession of the abilities (understanding, reasoning, and appreciation) needed to make financial decisions and choices that serve the individual's best interests’.^52^^(p.6)^ The development of three programme models focused on a person's individual context, were also informed by these legal criteria.^40,48,51^ For the FCAI, based on the ‘financial competency model’, multi-dimensional financial task assessment items were re-coded to evaluate these criteria.^47,48^ Darzins et al.'s^40^ six-step property/finance capacity assessment process explores these principles within a structured interview. The ‘financial decisional abilities model’^13^ and assessment tool, the Lichtenberg Financial Decision Rating Scale (LFDRS)^51^ focuses on an individual's financial decision-making ability for sentinel personal financial decisions. Appelbaum and Grisso's^49^ principles of understanding, appreciation, reasoning and expressing a choice are labelled as ‘intellectual factors’ within the model.^13,51^

Two models make a clear distinction of capacity being a legal framework, and financial management skills being a component of this.^37,53^ While not always explicitly referring to established criteria for legal capacity, seven models consider the difference of having capacity for specific financial tasks or decisions in contrast to overall financial capacity.^1–3,13,39,40,44,47,48,51,52,54^ Some of these models conceptualise overall financial capacity to be a sum of knowledge or ability in multiple separate financial domains.^1,3,44,54^ Others reason that overall financial capacity should consider an individual's context or real-world environment.^13,39,40,47,48,51,52^

*Sub-theme 2: Financial exploitation risk.* Three models focus specifically on financial decision-making ability and/or financial exploitation risk.^13,38,43,51^ Lichtenberg et al.^13^^(p.51)^ define ‘financial exploitation’ as ‘illegal or improper use of an older adult's funds or property for another person's profit or advantage’, with six associated domains including abuse of trust, theft and scams, coercion, financial entitlement, signs of possible financial abuse and money management difficulties. The accompanying model focuses on an individual's financial decision-making abilities. Within the model, ‘intellectual factors’ and long-standing personal values are considered alongside ‘contextual factors’ such as exposure to previous financial exploitation, financial situational awareness, psychological vulnerability and potential undue influence.^13,51^

Two models explore potential causal aspects of increased financial exploitation vulnerability, while also considering the risk related to loss of financial skills prior to a dementia diagnosis.^38,43^ A third model qualitatively explored the potential danger or risk related to the loss of financial skills prior to a dementia diagnosis from the perspective of people with dementia and their carers.^42^ Two recent models arising from qualitative research^55,56^ also consider self or carer-perceived increase in risk for financial exploitation vulnerability secondary to cognitive impairment from acquired brain injury.

#### Theme 3: Neuropathological cause of declining financial capability

This theme focused on the underlying neuropathological cause of declining financial capability. All three models aligned with this theme expressly focus on the neurological pathology of a decline in financial capability skills in people with mild cognitive impairment and Alzheimer's disease.^38,43,50^ Two of the models propose that changes in the brain underlie specific neurocognitive dysfunction, which drives functional decline in mild cognitive impairment and Alzheimer's disease.^43,50^ Copeland^50^ suggested that cortical thinning of the grey mantle in specific regions of interest contributes to a decline in everyday financial skills secondary to impairment in arithmetic (inferior parietal cortex), attention and executive functioning (precuneus/superior frontal cortex), and memory and learning (parahippocampal gyrus/entorhinal cortex). An imaging study provided ‘somewhat consistent’ support for this model's hypothesis.^50^ Spreng and colleagues^43^ hypothesised that structural and functional brain changes in social capacity neural pathways (default network brain regions, subcortical brain regions) and cognitive capacity neural pathways (lateral frontal and parietal brain regions) may lead to a decline in financial reasoning skills and increased social vulnerability, potentially escalating financial exploitation risk. A third model focused more on financial exploitation vulnerability being an early behavioural sign of underlying Alzheimer's disease neuropathology secondary to accumulation of β-amyloid in the default mode network (DMN).^38^ Fenton and colleagues^38^ theorised that the β-amyloid build-up results in structural and functional brain changes that could affect the connectivity between brain regions responsible for decision-making, value judgements and risk assessment.

### Confidence in the review findings

Assessment of confidence in the review findings was completed via application of GRADE-CERQual^41^ (Confidence in the Evidence from Reviews of Qualitative Research). Reviewers found high confidence in both theme 1 and 2, with no, or very minor concerns related to methodological limitations, coherence, adequacy and relevance. Although theme 3 generated no or very minor concerns regarding methodological limitations of related studies and coherence; minor concerns regarding adequacy were evident, with only 3 of the 21 included papers related to this theme. Moderate concerns were found regarding relevance as these 3 papers related only to people with dementia or mild cognitive impairment. As the review covered broader populations with acquired cognitive impairment, the overall GRADE-CERQual assessment of confidence in relation to theme 3 was downgraded to moderate confidence (see Supplementary Materials for: GRADE-CERQual assessment).

## Discussion

This review has focused on exploring and synthesising the qualitative evidence in relation to the conceptualisation of financial capability in adults with acquired cognitive impairment. Findings indicate that there are challenges related to consensus regarding conceptualisation of financial capability. Results revealed three key underlying themes in the literature, with the first addressing the multi-dimensionality of financial capability, and the second addressing financial decision-making ability and exploitation risk for legal capacity. Within both these themes, the focus of models and associated terminology displayed a shift over time from earlier independent knowledge, judgement or skill-based competency models to models that have a deeper consideration of environmental and personalised contextual factors. Several earlier published models are heavily focused on a person's ‘financial knowledge’ and individual ability to independently manage a range of financial tasks, mostly in a controlled clinical setting with hypothetical financial scenarios. Common terminology in these models include ‘financial capacity’ and ‘financial competence’, with both of these terms generally being utilised to represent an individual's financial task performance ability. Alternatively, some authors considered these models to be too focused on financial task completion rather than meeting the benchmarks for ‘legal capacity’ including the ability to understand and appreciate information and express a choice. Accordingly, models were generated with more concern for ‘financial judgement’ or ‘financial decision-making’ for sentinel financial decisions. Focus was also placed on identifying ‘financial exploitation risk’ when ‘financial decision-making’ may be compromised.

The third theme focusses on the underlying neuropathological cause of decline, specifically for people with mild cognitive impairment and Alzheimer's disease. However, alongside brain pathology, recent models have emphasised a person's ‘financial performance’ in individualised real-world settings, including social context and available supports, physical and sensory environmental aspects, economic context and availability of financial resources, and the impact of technology. Models considering these contextual factors have fuelled the rise of the adoption of economics-related terminology such as ‘financial capability’, whereby the emphasis has moved from financial independence to capability within an individual's particular context to meet financial needs and ensure ‘financial well-being’.

Conjecture exists in the literature regarding legal terminology, with both ‘financial competence’^1–3,44,54^ and ‘financial/legal capacity’^40,47,48,51^ used. ‘Legal capacity’ is preferred in Article 12 of the United Nations Convention on the Rights of Persons with Disabilities (UN-CRPD),^58^ with ‘financial competence’ the preferred term for the clinical assessment of ability to manage financial tasks in a controlled setting.^52^ While internationally accepted criteria for legal capacity are centred on principles of understanding information, appreciation of consequences and expression of choice;^49^ models have diverged in either considering this to be based upon a person's ability to make sentinel financial decisions^13,51^ or overall ability to manage financial affairs.^40^ The National Academies of Sciences^52^ advocates that both ‘financial competence’ and ‘financial performance’ in real-world environments can inform ‘financial capability’, acknowledging that a person may still be capable of managing financial affairs with support. Financial capability determinations should consider the consequences of restriction of personal autonomy against the protection of a person's best interest.^52^ This perspective aligns with emerging supported decision-making models of care for people with cognitive disability^52,59–62^ driven by the establishment of the UN-CRPD^58^ in relation to persons with disabilities equal rights to control their own financial affairs (Article 12.5),^58^ within the context of effective safeguards and independent review (Article 12.4).^58^ Therefore, clinicians making recommendations regarding financial capability that will be tested via legal proceedings may wish to consider a person's ability to understand information, appreciate consequences and express choice in regard to both overall financial competence or financial performance within a personal context, and with respect to specific noteworthy financial decisions.

Compared to ‘legal capacity’, the term ‘financial capability’ may resonate better for people who may experience improvement from rehabilitation or support. Recent rapid advancements of financial technology (‘Fintech’),^63^ and the digitisation of financial tasks, including the use of electronic financial instruments (EFI)^64^ has outdated some of the earlier models which view financial capability as a series of multidimensional tasks.^2,3,44,47,48^ Most importantly, there is no existing theoretical model to guide assessment practice concerning emerging technology use, including fraud or exploitation detection.^64,65^ However, the ‘Financial Management Activity Process’ (FMAP) is a process-based model that may provide guidance for acquired brain injury rehabilitation, accounting for technological change into the future by focusing on the activity process as relevant to an individual's context.^37^ Rehabilitation planning may also be enhanced by awareness of neurocognitive domains associated with financial capability for those with brain injury,^5,6,66^ dementia,^44,67^ and other neurological diseases.^68–72^

This review was limited to original versions or significant revisions of models developed in relation to the target population. Models that did not specifically focus on financial capability were excluded; therefore, broader models related to general cognitive, brain function or functional ability in which financial capability may have been a sub-component were not explored. While this review included programme models that provide direct guidance for assessment of financial capability, other tools for the assessment of financial capability which have been reviewed elsewhere^53,73^ did not meet our criteria for inclusion as a model. Nor were intervention studies included, with this being an area which requires future research.^74^ Four models were based on qualitative studies with people with acquired cognitive impairment.^37,42,55,56^ Continued consumer and carer involvement is essential in all aspects of future model, assessment or intervention development.

**Clinical messages**

- Financial competence is the preferred term for independent demonstration of financial skills, knowledge and judgement in a controlled setting.
- Financial competence alone does not determine financial capability, with consideration required of real-world financial performance within an individual's context. People may be capable of directing the management of finances with environmental and social supports in place.
- The terms ‘legal capacity’ or ‘financial capacity’ are increasingly reserved for legal judgements, which balance the right to autonomy with financial exploitation risk.
- The ‘Financial Management Activity Process’ (FMAP) model may provide guidance for financial capability rehabilitation for people with brain injury.

## Supplemental Material

sj-docx-1-cre-10.1177_02692155251347766 - Supplemental material for Conceptualisation of financial capability in adults with acquired cognitive impairment: A qualitative evidence synthesis Supplemental material, sj-docx-1-cre-10.1177_02692155251347766 for Conceptualisation of financial capability in adults with acquired cognitive impairment: A qualitative evidence synthesis by Sarah Swan, Freyr Patterson, Terra M Bredy and Jennifer Fleming in Clinical Rehabilitation

sj-docx-2-cre-10.1177_02692155251347766 - Supplemental material for Conceptualisation of financial capability in adults with acquired cognitive impairment: A qualitative evidence synthesis Supplemental material, sj-docx-2-cre-10.1177_02692155251347766 for Conceptualisation of financial capability in adults with acquired cognitive impairment: A qualitative evidence synthesis by Sarah Swan, Freyr Patterson, Terra M Bredy and Jennifer Fleming in Clinical Rehabilitation

sj-docx-3-cre-10.1177_02692155251347766 - Supplemental material for Conceptualisation of financial capability in adults with acquired cognitive impairment: A qualitative evidence synthesis Supplemental material, sj-docx-3-cre-10.1177_02692155251347766 for Conceptualisation of financial capability in adults with acquired cognitive impairment: A qualitative evidence synthesis by Sarah Swan, Freyr Patterson, Terra M Bredy and Jennifer Fleming in Clinical Rehabilitation

sj-docx-4-cre-10.1177_02692155251347766 - Supplemental material for Conceptualisation of financial capability in adults with acquired cognitive impairment: A qualitative evidence synthesis Supplemental material, sj-docx-4-cre-10.1177_02692155251347766 for Conceptualisation of financial capability in adults with acquired cognitive impairment: A qualitative evidence synthesis by Sarah Swan, Freyr Patterson, Terra M Bredy and Jennifer Fleming in Clinical Rehabilitation

sj-pdf-5-cre-10.1177_02692155251347766 - Supplemental material for Conceptualisation of financial capability in adults with acquired cognitive impairment: A qualitative evidence synthesis Supplemental material, sj-pdf-5-cre-10.1177_02692155251347766 for Conceptualisation of financial capability in adults with acquired cognitive impairment: A qualitative evidence synthesis by Sarah Swan, Freyr Patterson, Terra M Bredy and Jennifer Fleming in Clinical Rehabilitation
