# Supplementary material for: Conceptualisation of financial capability in adults with acquired cognitive impairment: A qualitative evidence synthesis
Source: Clin Rehabil. 2025 Jun 12;39(7):849–71. doi: 10.1177/02692155251347766 (PMC12198468; doi:10.1177/02692155251347766)
Supplement: sj-docx-1-cre-10.1177_02692155251347766 - Supplemental material for Conceptualisation of financial capability in adults with acquired cognitive impairment: A qualitative evidence synthesis [file sj-docx-1-cre-10.1177_02692155251347766.docx]

**Supplementary materials: Search Strategy**

**Conceptualisation of financial capability in adults with acquired cognitive impairment: A qualitative evidence synthesis**

**Full search terms**

|  | Conceptualisation | Financial capability | Acquired cognitive impairment | Filter |
| --- | --- | --- | --- | --- |
| MESH terms (PubMed) | ‘concept formation’  ‘models, psychological’  ‘occupational therapy’  ‘qualitative research’ | ‘decision making’  ‘financing, personal’  ‘financial management’  ‘mental competency/psychology’  ‘mental competency/legislation & jurisprudence’  ‘mental competency/standards’ | ‘aging’  ‘cognition’  ‘cognition disorders/ psychology’  ‘cognition disorders/rehabilitation’  ‘cognitive dysfunction/psychology’  ‘cognitive dysfunction/rehabilitation’  ‘dementia’  ‘geriatric assessment’  ‘brain injuries’  ‘brain injury, chronic/psychology’  ‘executive function’ | Adult: 19+ years  Young Adult: 19-24 years  Adult: 19-44 years  Middle Aged + Aged: 45+ years  Middle Aged: 45-64 years  Aged: 65+ years  80 and over: 80+ years |
| Key words (Title and Abstract Pubmed) | ‘concept formation’  ‘models, psychological’  ‘occupational therapy’  ‘qualitative research’  ‘theoretical framework’  ‘theoretical concept’  ‘theoretical model’  ‘conceptual framework’  ‘conceptual model’ | ‘decision making’  ‘financing, personal’  ‘financial management’  ‘mental competency/psychology’  ‘mental competency/legislation & jurisprudence’  ‘mental competency/standards’  ‘personal financing’  ‘financial well being’  ‘financial capability’  ‘financial capacity’  ‘financial competence’  ‘financial competency’  ‘financial management skills’  ‘financial decision making’  ‘financial performance’  ‘financial confidence’  ‘financial inclusion’  ‘financial literacy’  ‘financial exploitation’  ‘financial awareness’  ‘capacity assessment’  ‘informed decision making’  ‘money management’ | ‘aging’  ‘cognition’  ‘cognition disorders’  ‘cognitive dysfunction’  ‘cognitive impairment’  ‘dementia’  ‘geriatric assessment’  ‘brain injury’  ‘executive function’  ‘stroke’  ‘cerebrovascular accident’  ‘head injury’  ‘craniocerebral trauma’  ‘parkinson disease’  ‘parkinson’s disease’  ‘multiple sclerosis’  ‘cognitive function’  ‘impulsivity’ | Adult: 19+ years  Young Adult: 19-24 years  Adult: 19-44 years  Middle Aged + Aged: 45+ years  Middle Aged: 45-64 years  Aged: 65+ years  80 and over: 80+ years |

**Pub Med Full Search (MESH & T&A)**

**(((((('concept formation'[MeSH Terms]) OR ('models, psychological'[MeSH Terms])) OR ('occupational therapy'[MeSH Terms])) OR ('qualitative research'[MeSH Terms])) OR (((((((((('concept formation'[Title/Abstract]) OR ('models, psychological'[Title/Abstract])) OR ('occupational therapy'[Title/Abstract])) OR ('qualitative research'[Title/Abstract])) OR ('theoretical framework'[Title/Abstract])) OR ('theoretical concept'[Title/Abstract])) OR ('theoretical model'[Title/Abstract])) OR ('theoretical model'[Title/Abstract])) OR ('conceptual framework'[Title/Abstract])) OR ('conceptual model'[Title/Abstract]))) AND (((((((('decision making'[MeSH Terms]) OR ('financing, personal'[MeSH Terms])) OR ('financial management'[MeSH Terms])) ) OR ('mental competency/psychology'[MeSH Terms])) OR ('mental competency/legislation & jurisprudence'[MeSH Terms])) OR ('mental competency/standards'[MeSH Terms])) OR ((((((((((((((((((((((('decision making'[Title/Abstract]) OR ('financing, personal'[Title/Abstract])) OR ('financial management'[Title/Abstract])) OR ('mental competency/psychology'[Title/Abstract])) OR ('mental competency/legislation & jurisprudence'[Title/Abstract])) OR ('mental competency/standards'[Title/Abstract])) OR ('personal financing'[Title/Abstract])) OR ("financial well being"[Title/Abstract])) OR ('financial capability'[Title/Abstract])) OR ('financial capacity'[Title/Abstract])) OR ('financial competence'[Title/Abstract])) OR ('financial competency'[Title/Abstract])) OR ('financial management skills'[Title/Abstract])) OR ("financial decision making"[Title/Abstract])) OR ('financial performance'[Title/Abstract])) OR ('financial confidence'[Title/Abstract])) OR ('financial inclusion'[Title/Abstract])) OR ("financial literacy"[Title/Abstract])) OR ('financial exploitation'[Title/Abstract])) OR ('financial awareness'[Title/Abstract])) OR ('capacity assessment'[Title/Abstract])) OR ('informed decision making'[Title/Abstract])) OR ('money management'[Title/Abstract])))) AND (((((((((((('aging'[MeSH Terms]) OR ('cognition'[MeSH Terms])) OR ('cognition disorders/psychology'[MeSH Terms])) OR ('cognition disorders/rehabilitation'[MeSH Terms])) OR ('cognitive dysfunction/psychology'[MeSH Terms])) OR ('cognitive dysfunction/rehabilitation'[MeSH Terms])) OR ('dementia'[MeSH Terms])) OR ('geriatric assessment'[MeSH Terms])) OR ('brain injuries'[MeSH Terms])) OR ('brain injury, chronic/psychology'[MeSH Terms])) OR ('executive function'[MeSH Terms])) OR (((((((((((((((((('aging'[Title/Abstract]) OR ('cognition'[Title/Abstract])) OR ('cognition disorders'[Title/Abstract])) OR ('cognitive dysfunction'[Title/Abstract])) OR ('cognitive impairment'[Title/Abstract])) OR ('dementia'[Title/Abstract])) OR ('geriatric assessment'[Title/Abstract])) OR ("brain injury"[Title/Abstract])) OR ('executive function'[Title/Abstract])) OR ('stroke'[Title/Abstract])) OR ('cerebrovascular accident'[Title/Abstract])) OR ('head injury'[Title/Abstract])) OR ('craniocerebral trauma'[Title/Abstract])) OR ('parkinson disease'[Title/Abstract])) OR ('parkinson's disease'[Title/Abstract])) OR ('multiple sclerosis'[Title/Abstract])) OR ('cognitive function'[Title/Abstract])) OR ('impulsivity'[Title/Abstract])))**

Conceptualisation

| **(((('concept formation'[MeSH Terms]) OR ('models, psychological'[MeSH Terms])) OR ('occupational therapy'[MeSH Terms])) OR ('qualitative research'[MeSH Terms])) OR (((((((((('concept formation'[Title/Abstract]) OR ('models, psychological'[Title/Abstract])) OR ('occupational therapy'[Title/Abstract])) OR ('qualitative research'[Title/Abstract])) OR ('theoretical framework'[Title/Abstract])) OR ('theoretical concept'[Title/Abstract])) OR ('theoretical model'[Title/Abstract])) OR ('theoretical model'[Title/Abstract])) OR ('conceptual framework'[Title/Abstract])) OR ('conceptual model'[Title/Abstract]))** |  |
| --- | --- |

Financial Capability

**((((((('decision making'[MeSH Terms]) OR ('financing, personal'[MeSH Terms])) OR ('financial management'[MeSH Terms])) ) OR ('mental competency/psychology'[MeSH Terms])) OR ('mental competency/legislation & jurisprudence'[MeSH Terms])) OR ('mental competency/standards'[MeSH Terms])) OR ((((((((((((((((((((((('decision making'[Title/Abstract]) OR ('financing, personal'[Title/Abstract])) OR ('financial management'[Title/Abstract])) OR ('mental competency/psychology'[Title/Abstract])) OR ('mental competency/legislation & jurisprudence'[Title/Abstract])) OR ('mental competency/standards'[Title/Abstract])) OR ('personal financing'[Title/Abstract])) OR ("financial well being"[Title/Abstract])) OR ('financial capability'[Title/Abstract])) OR ('financial capacity'[Title/Abstract])) OR ('financial competence'[Title/Abstract])) OR ('financial competency'[Title/Abstract])) OR ('financial management skills'[Title/Abstract])) OR ("financial decision making"[Title/Abstract])) OR ('financial performance'[Title/Abstract])) OR ('financial confidence'[Title/Abstract])) OR ('financial inclusion'[Title/Abstract])) OR ("financial literacy"[Title/Abstract])) OR ('financial exploitation'[Title/Abstract])) OR ('financial awareness'[Title/Abstract])) OR ('capacity assessment'[Title/Abstract])) OR ('informed decision making'[Title/Abstract])) OR ('money management'[Title/Abstract]))**

Acquired cognitive impairment

**((((((((((('aging'[MeSH Terms]) OR ('cognition'[MeSH Terms])) OR ('cognition disorders/psychology'[MeSH Terms])) OR ('cognition disorders/rehabilitation'[MeSH Terms])) OR ('cognitive dysfunction/psychology'[MeSH Terms])) OR ('cognitive dysfunction/rehabilitation'[MeSH Terms])) OR ('dementia'[MeSH Terms])) OR ('geriatric assessment'[MeSH Terms])) OR ('brain injuries'[MeSH Terms])) OR ('brain injury, chronic/psychology'[MeSH Terms])) OR ('executive function'[MeSH Terms])) OR (((((((((((((((((('aging'[Title/Abstract]) OR ('cognition'[Title/Abstract])) OR ('cognition disorders'[Title/Abstract])) OR ('cognitive dysfunction'[Title/Abstract])) OR ('cognitive impairment'[Title/Abstract])) OR ('dementia'[Title/Abstract])) OR ('geriatric assessment'[Title/Abstract])) OR ("brain injury"[Title/Abstract])) OR ('executive function'[Title/Abstract])) OR ('stroke'[Title/Abstract])) OR ('cerebrovascular accident'[Title/Abstract])) OR ('head injury'[Title/Abstract])) OR ('craniocerebral trauma'[Title/Abstract])) OR ('parkinson disease'[Title/Abstract])) OR ('parkinson's disease'[Title/Abstract])) OR ('multiple sclerosis'[Title/Abstract])) OR ('cognitive function'[Title/Abstract])) OR ('impulsivity'[Title/Abstract]))**

|  | Conceptualisation | Financial capability | Acquired cognitive impairment | Filter |
| --- | --- | --- | --- | --- |
| MESH terms/subject headings (CINAHL) | “concept formation”  “models, psychological”  “occupational therapy”  “qualitative studies” | “decision making, patient”  “financial management”  “competency assessment” or  “competence (legal)” | “aging”  “cognition” or “cognition disorders” or “rehabilitation, cognitive” or “mild cognitive impairment”  “dementia or “frontotemporal dementia” or “dementia, vascular” or “dementia, multi-infarct”  “geriatric assessment”  “brain injuries”  “executive function” |  |
| Key words (Title and Abstract Cinahl) | “concept formation”  “psychological models”  “occupational therapy”  “qualitative research”  “qualitative study”  “theoretical framework”  “conceptual framework”  “theoretical concepts”  “theoretical model”  “conceptual model” | “decision making”  “financing, personal”  “financial management”  “mental competency”  “competency assessment”  “personal financing”  “personal financial management  “financial well being”  “financial capability”  “financial capacity”  “financial competence”  “financial competency”  “financial management skills”  “financial decision making”  “financial performance”  “financial confidence”  “financial inclusion”  “financial literacy”  “financial exploitation”  “financial awareness”  “capacity assessment”  “informed decision making”  “money management” | “aging”  “cognition”  “cognition disorders”  “cognitive dysfunction”  “cognitive impairment”  “dementia”  “geriatric assessment”  “brain injur*”  “executive function”  “stroke”  “cerebrovascular accident”  “head injur*”  “craniocerebral trauma”  “parkinson disease”  “parkinson’s disease”  “multiple sclerosis”  “cognitive function”  “Impulsivity” |  |

| MESH terms/subject headings (Embase) | ‘concept formation’  ‘psychological model’  ‘occupational therapy’  ‘qualitative research’  ‘qualitative study’  ‘theoretical framework’  ‘conceptual framework’  ‘theoretical model’  ‘conceptual model’ | ‘decision making’  ‘financial management’  ‘competency’  ‘competence’  ‘personal finance’  ‘personal financing’  ‘financing, personal’  ‘mental capacity’  ‘mental competency’  ‘personal financial management’  ‘economic well-being’  ‘financial well-being’  ‘financial abuse’  ‘financial exploitation’  ‘informed decision making’ | ‘aging’  ‘cognition’  ‘cognition disorders’  ‘cognitive defect’  ‘cognitive impairment’  ‘dementia’  ‘geriatric assessment’  ‘brain injury’  ‘executive function’  ‘stroke’  ‘cerebrovascular accident’  ‘head injury’  ‘craniocerebral trauma’  ‘parkinson disease’  ‘multiple sclerosis’  ‘cognitive function’  ‘impulsiveness’  ‘impulsivity’ |  |
| --- | --- | --- | --- | --- |
| Key words (Title and Abstract Embase) | ‘concept formation’  ‘psychological models’  ‘occupational therapy’  ‘qualitative research’  ‘qualitative study’  ‘theoretical framework’  ‘conceptual framework’  ‘theoretical concepts’  ‘theoretical model’  ‘conceptual model’ | Personal NEAR/4 ‘decision making’  Personal NEAR/4 ‘financ*’  ‘competenc* NEAR/10 financ*’  ‘competence’  ‘personal finance’  ‘personal financing’  ‘financing, personal’  ‘mental capacity’  ‘mental competency’  ‘competency assessment’  ‘personal financial management’  ‘economic well-being’  ‘financial well-being’  ‘financial capability’  ‘financial capacity’  ‘financial competence’  ‘financial competency’  ‘financial management skills’  ‘financial decision making’  ‘financial performance’  ‘financial confidence’  ‘financial inclusion’  ‘financial literacy’  ‘financial abuse’  ‘financial exploitation’  ‘financial awareness’  ‘capacity assessment’  ‘informed decision making’  ‘money management’ | ‘aging’  ‘cognition’  ‘cognitive defect’  ‘cognition disorders’  ‘cognitive dysfunction’  ‘cognitive impairment’  ‘dementia’  ‘geriatric assessment’  ‘brain injur*’  ‘executive function’  ‘stroke’  ‘cerebrovascular accident’  ‘head injury’  ‘craniocerebral trauma’  ‘parkinson disease’  ‘parkinson’s disease’  ‘multiple sclerosis’  ‘cognitive function’  ‘Impulsivity’  ‘impulsiveness’ |  |

| MESH terms (Psycinfo) | “concept formation”  “models, psychological”  “occupational therapy”  “qualitative research” | “decision making”  “financing, personal”  “financial management”  “mental competency/psychology”  “mental competency/legislation & jurisprudence”  “mental competency/standards” | “aging”  “cognition”  “cognition disorders/ psychology”  “cognition disorders/rehabilitation”  “cognitive dysfunction/psychology”  “cognitive dysfunction/rehabilitation”  “dementia”  “geriatric assessment”  “brain injuries”  “brain injury, chronic/psychology”  “executive function” |  |
| --- | --- | --- | --- | --- |
| Key words (Title and Abstract Psycinfo) | “concept formation”  “models, psychological”  “occupational therapy”  “qualitative research”  “theoretical framework”  “theoretical concept”  “theoretical model”  “conceptual framework”  “conceptual model” | “decision making”  “financing, personal”  “financial management”  “mental competency/psychology”  “mental competency/legislation & jurisprudence”  “mental competency/standards”  “personal financing”  “financial well being”  “financial capability”  “financial capacity”  “financial competence”  “financial competency”  “financial management skills”  “financial decision making”  “financial performance”  “financial confidence”  “financial inclusion”  “financial literacy”  “financial exploitation”  “financial awareness”  “capacity assessment”  “informed decision making”  “money management” | “aging”  “cognition”  “cognition disorders”  “cognitive dysfunction”  “cognitive impairment”  “dementia”  “geriatric assessment”  “brain injury”  “executive function”  “stroke”  “cerebrovascular accident”  “head injury”  “craniocerebral trauma”  “parkinson disease”  “Parkinson’s disease”  “multiple sclerosis”  “cognitive function”  “impulsivity” |  |

((((**title**: (“impulsivity”))) *OR* ((**abstract**: (“impulsivity”)))) *OR* (((**title**: (“cognitive function”))) *OR* ((**abstract**: (“cognitive function”)))) *OR* (((**title**: (“multiple sclerosis”))) *OR* ((**abstract**: (“multiple sclerosis”)))) *OR* (((**title**: (“parkinson’s disease”))) *OR* ((**abstract**: (“parkinson’s disease”)))) *OR* (((**title**: (“parkinson disease”))) *OR* ((**abstract**: (“parkinson disease”)))) *OR* (((**title**: (“craniocerebral trauma”))) *OR* ((**abstract**: (“craniocerebral trauma”)))) *OR* (((**title**: (“head injury”))) *OR* ((**abstract**: (“head injury”)))) *OR* (((**title**: (“cerebrovascular accident”))) *OR* ((**abstract**: (“cerebrovascular accident”)))) *OR* (((**title**: (“stroke”))) *OR* ((**abstract**: (“stroke”)))) *OR* (((**title**: (“executive function”))) *OR* ((**abstract**: (“executive function”)))) *OR* (((**title**: (“brain injury”))) *OR* ((**abstract**: (“brain injury”)))) *OR* (((**title**: (“geriatric assessment”))) *OR* ((**abstract**: (“geriatric assessment”)))) *OR* (((**title**: (“dementia”))) *OR* ((**abstract**: (“dementia”)))) *OR* (((**title**: (“cognitive impairment”))) *OR* ((**abstract**: (“cognitive impairment”)))) *OR* (((**title**: (“cognitive dysfunction”))) *OR* ((**abstract**: (“cognitive dysfunction”)))) *OR* (((**title**: (“cognition disorders”))) *OR* ((**abstract**: (“cognition disorders”)))) *OR* (((**title**: (“cognition”))) *OR* ((**abstract**: (“cognition”)))) *OR* (((**title**: (“aging”))) *OR* ((**abstract**: (“aging”)))) *OR* (((**MeSH**: (“executive function”)))) *OR* (((**MeSH**: (brain injury)))) *OR* (((**MeSH**: (“brain injuries”)))) *OR* (((**MeSH**: (“geriatric assessment”)))) *OR* (((**MeSH**: (“dementia”)))) *OR* (((**MeSH**: (“cognitive dysfunction”)))) *OR* (((**MeSH**: (cognition disorders)))) *OR* (((**MeSH**: (“cognition”)))) *OR* (((**MeSH**: (“aging”))))) *AND* ((((**title**: (“money management”))) *OR* ((**abstract**: (“money management”)))) *OR* (((**title**: (“informed decision making”))) *OR* ((**abstract**: (“informed decision making”)))) *OR* (((**title**: (“capacity assessment”))) *OR* ((**abstract**: (“capacity assessment”)))) *OR* (((**title**: (“financial awareness”))) *OR* ((**abstract**: (“financial awareness”)))) *OR* (((**title**: (“financial exploitation”))) *OR* ((**abstract**: (“financial exploitation”)))) *OR* (((**title**: (“financial literacy”))) *OR* ((**abstract**: (“financial literacy”)))) *OR* (((**title**: (“financial inclusion”))) *OR* ((**abstract**: (“financial inclusion”)))) *OR* (((**title**: (“financial confidence”))) *OR* ((**abstract**: (“financial confidence”)))) *OR* (((**title**: (“financial performance”))) *OR* ((**abstract**: (“financial performance”)))) *OR* (((**title**: (“financial decision making”))) *OR* ((**abstract**: (“financial decision making”)))) *OR* (((**title**: (“financial management skills”))) *OR* ((**abstract**: (“financial management skills”)))) *OR* (((**title**: (“financial competency”))) *OR* ((**abstract**: (“financial competency”)))) *OR* (((**title**: (“financial competence”))) *OR* ((**abstract**: (“financial competence”)))) *OR* (((**title**: (“financial capacity”))) *OR* ((**abstract**: (“financial capacity”)))) *OR* (((**title**: (“financial capability”))) *OR* ((**abstract**: (“financial capability”)))) *OR* (((**title**: (“financial well being”))) *OR* ((**abstract**: (“financial well being”)))) *OR* (((**title**: (“personal financing”))) *OR* ((**abstract**: (“personal financing”)))) *OR* (((**title**: (“mental competency”))) *OR* ((**abstract**: (“mental competency”)))) *OR* (((**title**: (“financial management”))) *OR* ((**abstract**: (“financial management”)))) *OR* (((**title**: (“financing, personal”))) *OR* ((**abstract**: (“financing, personal”)))) *OR* (((**title**: (“decision making”))) *OR* ((**abstract**: (“decision making”)))) *OR* (((**MeSH**: (“mental competency”)))) *OR* (((**MeSH**: (“financial management”)))) *OR* (((**MeSH**: (“financing, personal”)))) *OR* (((**MeSH**: (“decision making”))) *OR* ((**title**: (“decision making”))))) *AND* ((((**title**: (“conceptual model”))) *OR* ((**abstract**: (“conceptual model”)))) *OR* (((**title**: (“conceptual framework”))) *OR* ((**abstract**: (“conceptual framework”)))) *OR* (((**title**: (“theoretical model”))) *OR* ((**abstract**: (“theoretical model”)))) *OR* (((**title**: (“theoretical concept”))) *OR* ((**abstract**: (“theoretical concept”)))) *OR* (((**title**: (“theoretical framework”))) *OR* ((**abstract**: (“theoretical framework”)))) *OR* (((**title**: (“qualitative research”))) *OR* ((**abstract**: (“qualitative research”)))) *OR* (((**title**: (“occupational therapy”))) *OR* ((**abstract**: (“occupational therapy”)))) *OR* (((**title**: (“models, psychological”))) *OR* ((**abstract**: (“models, psychological”)))) *OR* (((**title**: (“concept formation”))) *OR* ((**abstract**: (“concept formation”)))) *OR* (((**MeSH**: (“qualitative research”)))) *OR* (((**MeSH**: (“occupational therapy”)))) *OR* (((**MeSH**: (“models, psychological”)))) *OR* (((**MeSH**: (“concept formation”)))))

|  | Conceptualisation | Financial capability | Acquired cognitive impairment | Filter |
| --- | --- | --- | --- | --- |
| MESH terms (Cochrane) | ‘concept formation’  ‘models, psychological’  ‘occupational therapy’  ‘qualitative research’ | ‘decision making’  ‘financing, personal’  ‘financial management’  ‘mental competency’ | ‘aging’  ‘cognition’  ‘cognition disorders’  ‘cognitive dysfunction’  ‘dementia’  ‘geriatric assessment’  ‘brain injuries’  ‘brain injury, chronic/psychology’  ‘executive function’ |  |
| Key words (Title and Abstract Cochrane) | ‘concept formation’  ‘models, psychological’  ‘occupational therapy’  ‘qualitative research’  ‘theoretical framework’  ‘theoretical concept’  ‘theoretical model’  ‘conceptual framework’  ‘conceptual model’ | ‘decision making’  ‘financing, personal’  ‘financial management’  ‘mental competency’  ‘personal financing’  ‘financial well being’  ‘financial capability’  ‘financial capacity’  ‘financial competence’  ‘financial competency’  ‘financial management skills’  ‘financial decision making’  ‘financial performance’  ‘financial confidence’  ‘financial inclusion’  ‘financial literacy’  ‘financial exploitation’  ‘financial awareness’  ‘capacity assessment’  ‘informed decision making’  ‘money management’ | ‘aging’  ‘cognition’  ‘cognition disorders’  ‘cognitive dysfunction’  ‘cognitive impairment’  ‘dementia’  ‘geriatric assessment’  ‘brain injury’  ‘executive function’  ‘stroke’  ‘cerebrovascular accident’  ‘head injury’  ‘craniocerebral trauma’  ‘parkinson disease’  ‘parkinson’s disease’  ‘multiple sclerosis’  ‘cognitive function’  ‘impulsivity’ |  |

ABI/INFORM

noft(“aging” or “cognition” or “cognition disorders” or “cognitive dysfunction” or “cognitive impairment” or dementia or “geriatric assessment” or “brain injur*” or “executive function” or stroke or “cerebrovascular accident” or “head injur*” or “craniocerebral trauma” or “parkinson disease” or “Parkinson’s disease” or “multiple sclerosis” or “cognitive function” or “impulsivity” ) – 1 228 982

personal NEAR/4 “decision making” or “financing, personal” or personal NEAR/4 “financial management” or “mental competency” or “competency assessment” or “personal financing” or “personal financial management” or “financial well being” or “financial capability” or “financial capacity” or “financial competence” or “financial competency” or “financial management skills” or “financial decision making” or “financial performance” or “financial confidence” or “financial inclusion” or “financial literacy” or “financial exploitation” or “financial awareness” or “capacity assessment” or “informed decision making” or “money management” – 2 289 040

noft(“concept formation” or “psychological models” or “occupational therapy” or “qualitative research” or “qualitative study” or “theoretical framework” or “conceptual framework” or “theoretical concepts” or “theoretical model” or “conceptual model” )

noft("concept formation" OR "psychological models" OR "occupational therapy" OR "qualitative research" OR "qualitative study" OR "theoretical framework" OR "conceptual framework" OR "theoretical concepts" OR "theoretical model" OR "conceptual model") AND noft(personal NEAR/4 "decision making" OR "financing, personal" OR personal NEAR/4 "financial management" OR "mental competency" OR "competency assessment" OR "personal financing" OR "personal financial management" OR "financial well being" OR "financial capability" OR "financial capacity" OR "financial competence" OR "financial competency" OR "financial management skills" OR "financial decision making" OR "financial performance" OR "financial confidence" OR "financial inclusion" OR "financial literacy" OR "financial exploitation" OR "financial awareness" OR "capacity assessment" OR "informed decision making" OR "money management") AND noft("aging" OR "cognition" OR "cognition disorders" OR "cognitive dysfunction" OR "cognitive impairment" OR dementia OR "geriatric assessment" OR "brain injur*" OR "executive function" OR stroke OR "cerebrovascular accident" OR "head injur*" OR "craniocerebral trauma" OR "parkinson disease" OR "Parkinson’s disease" OR "multiple sclerosis" OR "cognitive function" OR "impulsivity")

|  | Conceptualisation | Financial capability | Acquired cognitive impairment |
| --- | --- | --- | --- |
| Key words (Title and Abstract Scopus) | “concept formation”  “psychological models”  “occupational therapy”  “qualitative research”  “qualitative study”  “theoretical framework”  “conceptual framework”  “theoretical concepts”  “theoretical model”  “conceptual model” | Personal W/4 “decision making”  “financing, personal”  Personal W/4 “financial management”  “mental competency”  “competency assessment”  “personal financing”  “personal financial management”  “financial well being”  “financial capability”  “financial capacity”  “financial competence”  “financial competency”  “financial management skills”  “financial decision making”  “financial performance”  “financial confidence”  “financial inclusion”  “financial literacy”  “financial exploitation”  “financial awareness”  “capacity assessment”  “informed decision making”  “money management” | “aging”  “cognition”  “cognition disorders”  “cognitive dysfunction”  “cognitive impairment”  “dementia”  “geriatric assessment”  “brain injur*”  “executive function”  “stroke”  “cerebrovascular accident”  “head injur*”  “craniocerebral trauma”  “parkinson disease”  “parkinson’s disease”  “multiple sclerosis”  “cognitive function”  “Impulsivity” |
