# Supplementary material for: Conceptualisation of financial capability in adults with acquired cognitive impairment: A qualitative evidence synthesis
Source: Clin Rehabil. 2025 Jun 12;39(7):849–71. doi: 10.1177/02692155251347766 (PMC12198468; doi:10.1177/02692155251347766)
Supplement: sj-docx-2-cre-10.1177_02692155251347766 - Supplemental material for Conceptualisation of financial capability in adults with acquired cognitive impairment: A qualitative evidence synthesis [file sj-docx-2-cre-10.1177_02692155251347766.docx]

**Supplementary Materials: Extended version Table 2. Descriptive information for included papers, models and conceptual frameworks**

|  | Author/s professional background | Target Population | Theory Class-ification | Author (year), country of origin, type of publication | Study design /purpose | Definitions / Key concepts | Framework/model theoretical foundation and overview | Quality assess-ment rating |  |
| --- | --- | --- | --- | --- | --- | --- | --- | --- | --- |
| **1.**  **Who can decide? Property and Finance** | Geriatric Medicine | People with ‘question-able capacity’ | Programme model | Darzins, Molloy, Strang et al. (2000)^1^ Australia  Book | Outline of a six-step capacity assessment process for property /finance.  (Based on work completed in Canada on capacity assessment process for Government of Ontario.) | “**Capacity** is determined by whether individuals can understand and appreciate information, not whether they can perform tasks. Capacity and the lack of capacity are legal concepts. The definition of capacity is determined by the legal definition of capacity in each particular jurisdiction. It is determined by law and operationalised by the courts on the basis of cases. It is not determined by health care workers, except for certain, well-defined situations. The role of health care workers is limited to providing testimony as expert witnesses to the court.”^1(p2)^ | Developed via clinical experience of authors and iterative clinical use. Proposes 6 steps in assessing capacity to manage property including: (1) Valid trigger for assessment required; (2) Engage the person in the assessment process; (3) Gather facts to base assessment on including person’s situation, choices available and foreseeable consequences; (4) Educate person; (5) Capacity assessment, where structured interview is based on framework provided by a “decisional aid for capacity to manage property.”^1(p82)^ Decisional aid has eight domains including: knowledge of assets, debts, income, expenses/bills, obligations, knowing the problem, understanding choices, and appreciation of consequences of choices. Includes consideration of a person’s personal specific situation/financial affairs; (6) Act on assessment results (e.g. appointment of substitute decision-makers). | 4/6  Strong |  |
| **2.**  **Conceptual Model of Financial Capacity** | Neuropsychology (one author with dual Legal qualification) / Medicine | Alzheimer’s disease / Dementia | Programme model | Marson, Sawrie, Snyder, et al.  (2000)^2^  United States  Journal article | Development of a conceptual model and prototype instrument to investigate financial capacity in people with Alzheimer’s disease. | “**Financial capacity** represents a broad continuum of activities and specific skills. We conceptualized financial capacity as a series of discrete, clinically relevant domains of activity rather than as a unitary construct. A domain-based approach better approximates the multidimensionality of financial capacity and is consistent with the legal doctrine of limited **financial competency** adopted within most state legal jurisdictions, which recognizes that an individual may be competent to carry out some financial activities and not others.^3^”^(p878)^  “**Financial capacity** has been found to be an ‘advanced’ activity of daily life (along with using the telephone and eating), conceptually and statistically distinct from ‘household’ activities of daily life (i.e., meal preparation, shopping, and light and heavy housework) and ‘basic’ activities of daily life (i.e., bathing, dressing, walking, and toileting).^4^”^(p877)^ | Derived from author decision on included financial domains based on theoretical relevance to independent functioning, clinical relevance to health professionals, and general relevance to statutory criteria for financial competence. Model has two levels:  (1) General domains of financial activity that are of relevance to the independence of community dwelling older adults;  (2) Specific financial abilities or tasks that are relevant to individual domains and operationalize these domains. Includes 14 specific financial tasks within 6 domains: basic monetary skills, financial conceptual knowledge, cash transactions, checkbook management, bank statement management and financial judgement.  Forms basis of the Financial Capacity Instrument (FCI). | 2/6  Moderate |  |
|  |  |  |  | Model revision 1:  Earnst, Wadley, Aldridge, et al.  (2001)^5^  United States  Journal article | Revised model reviewed and approved by an expert panel (physicians, gerontologists, attorney, judge).  Revised model utilised to examine relationship between working memory and financial abilities in people with Alzheimer’s disease (AD). | “While progressive cognitive decline is a defining feature of AD, the relationship between such decline and loss of **financial capacity** in AD remains unclear. Research identifying cognitive correlates of declining financial abilities can enhance our scientific understanding of the neurological substrates of **financial competency** loss in dementia, and can inform clinical assessment of **financial skills** by identifying specific cognitive impairments associated with **financial competency** loss.”^(p109)^ | Revised model has three levels:  (1) Specific financial abilities (tasks);  (2) Broader financial activities (domains);  (3) Overall financial capacity (global).  Revised model includes:  19 financial tasks within 8 domains.  Two new domains (in addition to original 6) include bill payment (domain 7) and assets and estate arrangements (domain 8).  Introduction of two global scores including total score (domains 1-7) and domain 8 score (described as ‘experimental’).  Addition of 'core knowledge type' to tasks (declarative/ procedural/judgemental).  Basis of revised FCI. | 2/6  Moderate |  |
|  |  |  |  | Model revision 2:  Griffith, Belue, Sicola, et al.  (2003)^6^  United States  Journal article | Revised model presented in the context of use for assessment of financial capacity in people with mild cognitive impairment (MCI). | “**Financial capacity** is a higher order ADL that is critical to independent functioning^7^ and very sensitive to mild AD.^2, 5, 8^ In studying functional change in MCI, therefore, **financial knowledge** and **skills** have proven to be a good place to focus.^9, 10^”^(p449)^ | Revised model also has three levels and includes 18 financial tasks within 9 domains.  One additional domain since previous revision being Investment decision-making (domain 9). This domain consists of task previously incorporated into domain 6 regarding Financial Judgment.  Two global scores (domains 1-7; 1-8).  Domain 9 not included in overall score (due to irrelevance to some).  Addition of ‘difficulty’ classification to tasks (simple/complex).  Basis of revised FCI. | 2/6  Moderate |  |
|  |  |  |  | Model reframed as:  Clinical model: Financial capacity as financial skills relevant to independence  Marson (2016)^11^  United States  Journal article | Overview of conceptual models of financial capacity, and guidance of assessment processes. | “The ability independently to manage one’s financial affairs, known as **financial capacity**, is an everyday life skill of critical importance.^12-15^ **Financial capacity** is tied closely to personal independence and successful function in the community, and has been equated in importance to ‘diet, exercise, and sleep’ as a key indicator of overall health status in adults.^16^”^(p541)^  “**Financial capacity** can also be conceptualized as a broad set of clinically relevant  **financial skills** and **activities** necessary for independent function in the community.”^(p543)^ | Model essentially unchanged from 2003 revision, however titled differently to reflect an extension of early IADL assessment proposed by Lawton & Brody^17^ that views financial capacity as financial skills and judgements relevant to independent living. | 3/6  Moderate |  |
| **3.**  **Financial Competency Model** | Psychology | Adults with cognitive impairment | Working model | Webber, Reeve, Kershaw, et al. (2002)^18^ Australia  Journal article | Early development of model briefly described based on literature review and survey. | “In sum, the legal definition of **financial competence** in Australia focuses on a person’s ability to make rational decisions about his or her money and assets.”^(p250)^  “A valid assessment of **financial competence** is needed both to protect the rights of people who are competent and also to protect those who may be at risk of **financial exploitation.**”^(p248)^  “One of the responsibilities of the guardianship tribunal or court is to decide whether individuals lack the **capacity** to look after their own financial affairs.^19^ It is important to note that the determination of ‘**incompetence**’ is a legal and not a psychological decision because the court or tribunal is ultimately responsible for determining whether a person is **competent**.^3^”^(p249)^ | Brief description of model based on 63 fiscal skills derived from literature review that were then rated by several groups of professionals (*n* = 64) (psychologists, medical practitioners and lawyers).  Factor analysis indicated 4 dimensions of importance: financial judgement, everyday financial abilities, estate management, debt management. | 1/6  Moderate |  |
|  |  |  | Conceptual framework | Model revised: Kershaw & Webber  (2004)^20^  Australia  Journal article | Further model development based on factor analysis of a wider survey of the importance of financial tasks and skills for financial competence applicable to a scenario of an adult with cognitive impairment | “..there is no universally accepted definition of ‘**competence**.’^21^ The terms ‘**competency**’ and ‘**capacity**’ have each been used interchangeably throughout the literature^22^; however, as pointed out by Marson,^8^ these two terms are ‘substantively different.’^(p. 1)^”^(p338)^  “Willis^23^ reported that although psychological definitions focus on ‘**competence**’, legal definitions focus more on **‘incapacity’**. In this article, we use the term **‘competence’** to refer to the ability to adequately carry out specific tasks related to a domain, and we acknowledge where others use the term ‘capacity.’” ^(p338-339)^  “**Financial competency** is a broad term encompassing much more than skills and abilities, and any competency/incompetency decision should involve the evaluation of many different aspects of the individual being assessed, such as diagnosis and context.”^(p348)^ | Derived from survey of professionals (e.g., lawyers, medical practitioners, psychologists) and university students (*n*=212) rating the importance of financial tasks and skills for financial competence in relation to a scenario of a person with cognitive impairment.  Four dimensions identified by Webber et al.,^18^ supported by larger survey, with a further two dimensions identified.  Consists of 6 dimensions that emerged from factor analysis of 61 original items including: everyday financial abilities, financial judgement, estate management, debt management, cognitive ability (basic literacy and numeracy skills) and support resources (assistance provided by others and the individual’s assistance-seeking skills).  All six dimensions significantly correlated with each other and explained more than 54% of the total variance. | 4/6  Strong |  |
|  |  |  | Programme model | Model evaluation:  Kershaw & Webber (2008)^24^ Australia Journal article | Development and validation of the Financial Competence Assessment Inventory (FCAI). | “..**financial competence** may be defined differently by different people and may take into account different aspects of **financial competence** from one person to the next.”^(p40)^  “..the important dimensions of **financial competence** included everyday financial abilities (e.g., paying bills) financial judgement (e.g., financial goals), cognitive functioning related to financial tasks (e.g., functional memory), estate management (e.g., understanding Power of Attorney), debt management, and support resources (e.g., knowing where to look for help in managing finances.^20^ Use of a multidimensional model makes it theoretically possible to examine individual differences in **financial competence**..”^(p. 41)^ | Model of financial competence was utilised to develop the Financial Competence Assessment Inventory (FCAI). It has a structured interview format with 41 questions/tasks related to financial abilities.  Results provided supporting evidence that the six-dimension model of financial competence measured by the FCAI can be useful in assessing the financial competence of adults following cognitive impairment.  FCAI items were also recoded and used to evaluate the Appelbaum and Grisso^25^ four legal criteria ‘understanding’, ‘appreciation’, ‘reasoning’, and ‘‘expressing a choice’’. | 4/6  Strong |  |
| **4.**  **Working model of financial capacity** | Clinical psychology / Neuropsychology (dual Legal qualification) | Older adults, including those with Alzheimer’s disease. | Working model | Moye & Marson (2007)^26^  United States  Journal article | Overview of existing research related to medical decision-making capacity and financial capacity. | “We use the term **capacity** to refer to a dichotomous (yes or no) judgment by a clinician or other professional as to whether an individual can perform a specific task (such as driving or living independently) or make a specific decision (such as consenting to health care or changing a will)...Two of these require a broad set of cognitive and procedural skills, independent living and **general financial management**.”^(p3)^  “Decisions about **capacity** are ultimately legal judgments enforced by the power of the state. However, in practice, the majority of determinations of diminished **capacity** are probably made outside of the courtroom, by clinicians, attorneys, adult protective service workers, and other professional groups working with the elderly population…situations requiring guardianship or conservatorship are resolved in a court of law, and they require a legal determination regarding **competency**.”^(p. 3)^  “**Financial capacity** comprises a broad range of conceptual, pragmatic, and judgment abilities that are critical to the independent functioning of adults in our society^2, 27^…**Financial capacity** is an ‘advanced’ activity of daily living (ADL), also called an instrumental ADL or IADL^4^...are mediated by higher cognitive function.”^(p7)^ | Model combines clinical and cognitive neuropsychological aspects, highlighting the contribution of a person’s declarative knowledge, procedural knowledge and judgement abilities.  (1) Declarative knowledge includes a person’s ability to outline details regarding personal finances, and general financial facts such as value of currency and meaning of terms such as loan and debt;  (2) Procedural knowledge is related to learned motor-based financial skills including how to use an ATM, count change, write cheques, complete simple online banking tasks;  (3) Judgement-based abilities include the utilisation of declarative and procedural financial knowledge and skills to make financial judgements in the best self-interest of the person. | 2/6  Moderate |  |
|  |  |  |  | Model reframed as Cognitive Psychological Model: Financial capacity as types of financial knowledge.  Marson (2016)^11^  United States  Journal article | Overview of conceptual models of financial capacity, and guidance of assessment processes. | “The ability independently to manage one’s financial affairs, known as **financial capacity,** is an everyday life skill of critical importance.^12-15^ **Financial capacity** is tied closely to personal independence and successful function in the community, and has been equated in importance to “diet, exercise, and sleep” as a key indicator of overall health status in adults.^16^”^(p541)^ | Model essentially unchanged from original publication, however titled differently to acknowledge cognitive psychological basis.  Further elaboration on processes and relationships involved in declarative knowledge, procedural knowledge and judgement. | 3/6  Moderate |  |
| **5.**  **Conceptual Model of Declining Financial Capacity in amnestic mild cognitive impairment (MCI) and Alzheimer’s disease.** | Neuropsychology | Alzheimer’s disease (AD) and amnestic mild cognitive impairment (aMCI) | Causal model | Copeland  (2013)^28^  United States  PhD Dissertation  Supervisors:  Triebel & Marson. | Doctoral dissertation with model linked to imaging study that had ‘somewhat consistent’ results to hypotheses. | “**Financial capacity** encompasses an array of conceptual, pragmatic, and judgement abilities, ranging from relatively simple knowledge and skills such as counting and naming currency to more complex knowledge and skills of bank statement management and paying bills^2^….**Financial capacity**, driving, and medication management are all cognitively mediated complex IADLs that can easily be distinguished from both household and basic ADL skills.^2, 4^”^(p8)^ | Neuropathological changes that occur in the brain in people with aMCI and AD (cortical thinning of the grey mantle in specific regions of interest) underlie specific neurocognitive impairment in arithmetic (inferior parietal cortex), attention and executive function (precuneus/superior frontal cortex) and memory and learning (parahippocampal gyrus/entorhinal cortex) that contribute to a decline in everyday financial skills. | 6/6  Strong |  |
| **6.**  **Financial Decisional Abilities Model** | Clinical Psychology / Neuropsychology / Clinical Geropsychology / Gerontology | Older adults, including those with cognitive impairment / dementia. | Conceptual framework / Programme model | Lichtenberg, Stoltman, Thicker, et al. (2015)^29^  United States  Journal article | Literature review derived proposed conceptual model with focus on general decisional abilities and financial exploitation.  Model then refined by two groups of experts.  Assessment instrument based on model outlined: Lichtenberg financial decision rating scale (LFDRS) with 5 example case studies. | **Financial exploitation**: “illegal or improper use of an older adult’s funds or property for another person’s profit or advantage”^30^ with six domains: theft and scams, abuse of trust, financial entitlement, coercion, signs of possible financial abuse, and money management difficulties.^(p51)^  **Financial capacity**: “the ability to manage money and financial assets in ways consistent with one’s values or self-interest,”^31^ unless evidence to the contrary has been confirmed.^(p53)^  “While the **financial-exploitation** literature has focused on risk factors for financial abuse and definitions for financial exploitation, the **financial capacity** literature has emphasized financial knowledge and skills and, to a lesser extent, financial judgment. Yet in the context of a specific financial decision, it is essential to determine whether the older adult’s judgment is authentic and the integrity of his or her **financial decisional abilities** intact.”^(p56)^ | Founded on person-centred approach (whole person dementia assessment model^32^) with focus on literature relating to decisional abilities and financial decision-making; and Appelbaum & Grisso decision making model.^25^  Refined by two groups of experts: health professionals (*n*= 6, psychologists/psychiatrists) and people working directly with older adults (*n*=12, law enforcement, bank personnel, protective services, financial planners, lawyers).  Encompasses contextual factors and intellectual factors, and whether there is consistency with a person’s values.  Contextual factors include financial situational awareness, past financial exploitation, psychological vulnerability, and undue influence.  Intellectual factors encompass functional abilities to make a choice, explain rationale, understand, and appreciated relevant factors involved. | 6/6  Strong |  |
|  |  |  | Programme model | Later referred to as: Financial Decisional Capacity Model  Lichtenberg, Ocepek-Welikson, Ficker, et al. (2018)^33^  United States  Journal article | Examined empirical support for conceptual model, and psychometric properties of the related measurement scale, the Lichtenberg Financial Decision Rating Scale (LFDRS). | “Most **financial capacity** measures include a number **of financial domains**, such as bill paying, checkbook management, and cash transactions,^8^ yet the legal standards for **financial incapacity** are strongly related to informed (**financial) decision making**. The rating scale we present here was created to measure clinical judgment of **capacity** (i.e., capacity for a specific decision or transaction).”^(p2)^  “**Financial capacity** as applied to this paper is the capacity for financial transactions as applied to legal standards (i.e. a new rating scale that measures informed financial decision making for actual decisions/transactions).”^(p2)^  “**Financial decision making** is emerging as a separate construct from cognition and from **financial management skills**.”^(p4)^ | Model re-titled and study results confirmed the reliability of the LFDRS and supported the conceptual model.  LFDRS can be utilised for clinical assessment of real-world financial decision-making and capacity.  LFDRS is unique in focusing on actual financial decisions with consideration of contextual variables.  Model and assessment evaluate not only the impact of cognition on financial decision-making abilities, but also the contribution of financial situational awareness and psychological vulnerability. | 6/6  Strong |  |
| **7.**  **Social Cognitive Neuroscience Model for Assessing Financial Exploitation Risk** | Psychology / Neuroscience / Neurology | Older adulthood, mild cognitive impairment & dementia. | Causal model / Conceptual framework / Programme model | Spreng, Karlawish & Marson.  (2016)^34^  United States  Journal article | Literature review derived novel framework and assessment tool of financial exploitation risk in older adulthood following age-related changes in the brain, cognition, and social functioning. | “**Financial exploitation** is a common form of elder mistreatment^35, 36^”^(p. 320)^…“Recent studies have identified individual, or person-centred, risk factors for **financial exploitation**. Foremost among these is reduced cognitive functioning in older adulthood^37^ and mild cognitive impairment.^38^”^(p321)^  “These findings, as well as investigations of **financial capacity** in aging and brain disease,^2, 39^ suggest that cognitive changes associated with aging and brain disease may impair **decision-making abilities**, leading to heightened financial vulnerability in these populations.^40^”^(p322)^ | Focuses on person-centred factors such as functional and neuropsychological capacities in context of age-related changes in brain structure and function.  Two neurally and behaviourally distinct interacting pathways (cognitive capacity & social capacity) are associated with structural and functional brain changes that may increase financial exploitation risk.  Changes in cognitive capacity associated with a decline in financial skills and possible increase in exploitation due to financial mismanagement (decline in fluid reasoning ability due to changes in lateral frontal and parietal brain regions).  Changes in social cognition increase vulnerability to financial exploitation via social influence, deception, or coercion (decline in social capacity due to changes in default network brain regions, or their interactions with affect processing subcortical brain regions). Basis of the Financial Competence in Everyday Decision-making (FCED) assessment tool. | 6/6  Strong |  |
| **8.**  **Institute of Medicine Conceptual Model of Financial Capability** | Psychiatry / Neurology / Medicine / Public Health / Social Work / Neuropsychology / Occupational Therapy | Adults with disabilities (including those with cognitive impairment) | Conceptual framework | National Academies of Sciences, Engineering and Medicine  (2016)^41^  United States  Book | Conceptual model proposed within an evaluation of the Social Security Administration’s (SSA’s) capability determination processes for adult beneficiaries in the United States. | **Financial capability**: “The management or direction of the management of one’s funds in a way that routinely meets one’s best interests.”^(p5)^  **Financial performance**: “An individual’s degree of success in handling financial demands in the context of the stresses, supports, contextual cues, and resources in the individual’s actual environment.”^(p6)^  **Financial competence**: “The financial skills one possesses, as demonstrated through financial knowledge and financial judgment, typically assessed in a controlled (e.g., office or clinical) setting.”^(p6)^  **Financial judgement:** “Possession of the abilities (understanding, reasoning, and appreciation) needed to make financial decisions and choices that serve the individual’s best interests.”^(p6)^  **Financial knowledge**: “Possession of the declarative knowledge (i.e., information that a person knows) and procedural knowledge (i.e., knowing how to perform a task) required to manage one’s finances (e.g., the concept of money, values of currency, making change, check writing, use of automatic teller machines, and online banking procedures.”^(p6)^  “In the committee’s model, **financial literacy** would be most akin to financial knowledge.”^(p27)^ | Derived from literature review, review of other capability determination processes, and multidisciplinary expert consensus.  Based on the importance of individual autonomy in decision-making; with reference to the International Classification of Functioning (ICF) in regard to conceptualising disability in terms of the interaction between a person’s functional capacity and their individual environment.  Promotes clear definitions of concepts related to financial capability, and their relationships.  Makes the distinction between financial competence and financial performance. While financial skills, knowledge and judgement contribute to financial competence; financial performance also includes additional contextual factors such as stresses, supports, cues or resources in a person’s actual environment (real-world functioning).  Acknowledges that a person may be financially competent in a controlled environment, but that this may not extend to a real-world environment; or that conversely someone who is not financially competent in a controlled environment may demonstrate appropriate financial performance with support systems or assistance in place, therefore being financially capable in a real-world environment. | 6/6  Strong |  |
| **9.**  **Concept of financial management** | | Occupational  Therapy / Neuropsychology | Acquired cognitive impairment | Working model | Engel, Bar, Beaton, et al.  (2016)^42^ Canada  Journal article | Systematic review that outlines the concept of financial management and identifies instruments that quantify financial management skills in adults with acquired cognitive impairment. | “**Financial management** represents a complex set of skills and judgments that can be difficult to comprehensively measure.^2, 43^ In this review we use “**financial management** **skills**” to connote both skills and judgments required in this area, and embrace a multidimensional concept that can be measured through observable and non-observable characteristics.^44^ **Financial management skills** have also been called money management skills, financial capacity, financial competency, or financial literacy; they encompass a wide range of abilities needed to handle one’s money and manage one’s property.^2, 8, 20, 35, 43, 45-47^ ”^(p77)^ | Included as a novel conceptual definition.  Financial management skills outlined as encompassing multiple levels of functioning in relation to the International Classification of Functioning, Disability and Health (body functions/mental functions, activity and participation).  Distinction is made between financial management skills and the legal construct of financial capacity/competency, which is decided in a court of law. Financial management skills assessment promoted as a component of capacity/competency assessment. | 3/6  Moderate |
| **10.**  **Four-factor structure of financial capacity** | | Neuropsychology (one author with dual Legal qualification) / Medicine | Older adulthood, mild cognitive impairment & dementia. | Working model | Gerstenecker, Triebel, Eakin, et al. (2018)^48^ United States  Journal article | Proposed model based on exploration of the factor structure of financial capacity.  . | “**Financial capacity** is a medical–legal construct that represents the ability to independently manage one’s own financial affairs in a manner consistent with personal self-interest and  values.^13, 14^ Along with driving and mobility, **financial capacity** is a core aspect of individual autonomy in our society.^8, 14, 49^ **Financial capacity** involves not only performance skills (e.g., counting coins/currency accurately, completing a check register accurately, paying bills) but also judgment skills that promote financial self-interest, and values that guide personal financial choices.”^(p. 2)^ | Factor structure of financial capacity explored by using a performance-based assessment (Financial Capacity Instrument) as a proxy for the construct.  Four extracted factors identified to be core components and accounted for 46% of the variance. The four factors included:  (1) Basic monetary knowledge and calculation skills, including semantic knowledge of currency values and arithmetic calculation skills (accounted for 35.8% of shared variance);  (2) Financial judgement, comprising items targeted at ability to detect and avoid fraudulent ‘scams’ on the telephone/via mail, (accounted for 5.2% of shared variance);  *(*3) Financial conceptual knowledge, consisting of items ascertaining comprehension of simple financial concepts such as knowing what a debt is and reasons for banking money etc. (accounted for 2.6% of shared variance);  *(*4) Financial procedural knowledge, related to ability to write a cheque/record a transaction in a cheque register (accounted for 2.6% of shared variance). | 5/6  Strong |
| **11.**  **The Financial Management Activity Process (FMAP)** | | Occupational Therapy / Neuropsychology | Acquired brain injury (ABI) | Conceptual framework | Engel, Beaton, Green, et al.  (2019)^50^ Canada  Journal article | Conceptual framework to guide ABI rehabilitation derived from grounded theory qualitative study regarding the experiences, actions and processes of adults living with ABI and close others who assist them with financial management tasks. | “Finances are ubiquitous to life; monetary capital is required to satisfy basic needs, accomplish everyday activities, and participate in chosen life roles. Thus, to meet one’s current and future needs and wants, adults must participate in **financial management** (FM) occupations, such as paying for goods and services, budgeting available resources, and saving for the future. Managing one’s finances is a central principle to North American adult life and shapes one’s autonomy and independence in other chosen life activities and roles.^51, 52^”^(p197)^ | Derived from constructivist grounded theory, based on theoretical perspectives of symbolic interactionism and social constructivism.Framework has three key phases that are fluid, with dynamic interrelated interactions:  (1) Identifying a financial management activity that requires completion;  (2) Exploration of ‘filter factors’ related to the person, environment or activity that may influence task completion;  (3) Personalizing process, whereby trusted strategies and processes are used to facilitate task completion including simplifying and organising; recording and checking records; scheduling; setting personalised financial management policies; using or directing assistance; and obtaining advice or knowledge. Strategies and processes are applied within a ‘lens of trust’, whereby judgements are made regarding the ability to trust themselves, others, technology, and organisations (such as banks, financial services personnel etc.).  A variety of technology options identified to assist in strategy application including low-technology (pencil/paper) and high technology (smartphones, internet etc.), allowing the framework to remain relevant as technology advances. | 6/6  Strong |
| **12.**  **Financial exploitation vulnerability manifests as an early behavioural sign of underlying Alzheimer’s disease related neuro-pathology.** | | Psychology / Medicine / Gerontology | Adults at risk / or who have Alzheimer’s dementia | Causal model | Fenton, Weissberger, Boyle, et al.  (2022)^53^  United States  Journal article | Literature review derived model based on review of neuroimaging, neuropathological, and cognitive correlates identified as underlying financial exploitation vulnerability in individuals at risk of cognitive decline. | “We use the term **financial exploitation** to encompass instances when an older adult’s resources are improperly used by a trusted other for the benefit of someone other than the older adult. We include financial fraud and scams in this definition because the older adult has a presumption of trust, although the scammer may be previously unknown to the victim.^54^”^(p2)^ | Proposes that early accumulation of Alzheimer’s disease related neuropathology (β-amyloid within the default mode network [DMN]) and resulting structural/functional brain changes (neural connectivity disruption between brain regions involved in decision-making, risk assessment, value judgements) manifest in increased financial exploitation vulnerability that is seen in subtle changes in cognition and impaired decision-making. | 3/6  Moderate |
| **13.**  **The digitisation of financial management skills in dementia since Covid-19** | | Psychology / Carers of people with dementia | Dementia | Working model | Giebel, Halpin, Tottie, et al. (2023)^55^  United Kingdom  Journal article | A qualitative interview study exploring the effect of the COVID-19 pandemic and digitalisation of financial management skills in people with dementia and their carers. | “**Financial management skills** are reported to be the first instrumental activity of daily living (IADL) to deteriorate in dementia…up to 10 years prior to a diagnosis.^56^ Subsequent research has further shown how the initiative, not only the performance, to engage in financial management tasks deteriorates early on in dementia,^57^ with wider finance management abilities linked to executive functioning^58^….**Financial management**, or **financial capacity** as coined by some, can be broken down into individual tasks.”^(p. 2)^ | Qualitative descriptive study resulting in five overarching themes (with subthemes) including: (1) Potential dangers of early loss of finance management skills; (2) Face-to-face shopping experiences and skills (lack of support and recognition from staff and other shoppers, feeling anxious and rushed, maintaining independence or supporting someone to be); (3) Barriers and facilitators of moving to digital (online use benefits/disadvantages); (4) COVID-19 triggered fast-tracked digitisation; (5) Carer impact due to supporting someone with finances (additional caring responsibilities, acceptance from person with disability, no support available). | 5/6  Strong |
| **14. Contextual factors of financial capability** | | Occupational  Therapy / Neuropsychology / Nursing / Psychology / Public Health / Social work | Acquired brain injury | Working model | Engel, Arowolo, Ewesesan, et al. (2024)^59^  Canada  Journal article | A qualitative photovoice study to identify financial capability and financial well-being contextual factors. | “Economic daily functioning is very important to human health and well-being and includes both the concepts of (i) **financial capability**, which is defined as the knowledge/literacy, skills, attitudes, and applied behaviors related to managing money, accessing financial resources, planning ahead financially, making financial choices, and getting financial related help,^60-62^ and (ii) **financial well-being**, which is defined as an individual’s financial outcomes including their subjective feelings of financial stress and objective ability to meet current and future financial needs.^61^”^(p273)^ | Qualitative descriptive study about contextual factors (barriers and facilitators) related to financial capability and financial well-being following ABI. Four themes of contextual factors generated including: (1) Economic context (finding adequate financial resources/income, making sense of complex financial information and process); (2) Social context (having a trusted person to provide assistance/guidance, bias and stigma related to ‘invisible’ disability); (3) Physical and sensory environment (layout and design of financial institutions); (4) Technology environment (benefits and challenges). | 5/6  Strong |
| **15. Financial capability and financial well-being of adults with ABI** | | Occupational Therapy / Computer science / Nursing / Psychology / Public Health / Social work | Acquired brain injury | Working model | Engel, Ewesesan, Arowolo, et al. (2024)^63^  Canada  Journal article | A pilot survey of adults with ABI and close others to examine financial capability and financial well-being experiences and challenges. | “**Financial capability** **(FC)** is the knowledge, skills, attitudes/confidence, and applied behaviours related to managing money, accessing financial resources, planning and making choices related to finances, and securing financial-related help when needed.^60, 61^”^(p2)^  “**Financial well-being** **(FWB)** is a larger concept that encompasses **FC**, where **FWB** is the subjective or objective outcomes of financial or economic-related behaviours and activities within a socioeconomic context and influenced by other physical, sensory, and technological environmental factors.^50, 61^ ^(p2)^ | Cross-sectional survey produced quantitative frequency data and narrative data from an open-ended question about managing finances after ABI. Four categories were developed from coding of the narrative responses about FC or FWB after ABI including: (1) Complexity of FC (including lack of support services); (2) Accessing and navigating FWB resources (resource availability, eligibility and access issues); (3) Increased financial exploitation vulnerability (perceived risk of abuse or fraud); (4) Implications on FWB from downward shift in income and employment (economic effect of job/income loss). | 5/6  Strong |

1. Darzins P, Molloy DW, Strang D, et al. *Who can decide? : the six step capacity assessment process* Adelaide, South Australia.: Memory Australia Press, 2000.

2. Marson DC, Sawrie SM, Snyder S, et al. Assessing financial capacity in patients with alzheimer disease: a conceptual model and prototype instrument. *Arch Neurol* 2000; 57: 877-884. DOI: 10.1001/archneur.57.6.877.

3. Grisso T. *Evaluating Competencies: Forensic Assessments and Instruments* New York, NY: Plenum Press, 1986.

4. Wolinsky FD and Johnson RJ. The use of health services by older adults. *J Gerontol* 1991; 46: S345-S357. DOI: 10.1093/geronj/46.6.S345.

5. Earnst KS, Wadley VG, Aldridge TM, et al. Loss of financial capacity in alzheimer's disease: the role of working memory. *Aging Neuropsychol Cogn* 2001; 8: 109-119. DOI: 10.1076/anec.8.2.109.839.

6. Griffith HR, Belue K, Sicola A, et al. Impaired financial abilities in mild cognitive impairment: a direct assessment approach. *Neurology* 2003; 60: 449-457. DOI: 10.1212/WNL.60.3.449.

7. Melton G, Petrila J, Poythress N, et al. *Psychological evaluations for the courts*. New York: Guilford Press, 1987.

8. Marson DC. Loss of financial competency in dementia: conceptual and empirical approaches. *Aging Neurospsychol Cogn* 2001; 8: 164-181. DOI: 10.1076/anec.8.3.164.827.

9. Daly E, Zaitchik D, Copeland M, et al. Predicting conversion to Alzheimer disease using standardized clinical information. *Arch Neurol* 2000; 57: 675-680. DOI: 10.1001/archneur.57.5.675.

10. Tabert MH, Albert SM, Borukhova-Milov L, et al. Functional deficits in patients with mild cognitive impairment: prediction of AD. *Neurology* 2002; 58: 758-764. DOI: 10.1212/wnl.58.5.758.

11. Marson DC. Conceptual models and guidelines for clinical assessment of financial capacity. *Arch Clin Neuropsychol* 2016; 31: 541-553. DOI: 10.1093/arclin/acw052.

12. Marson DC. Clinical and ethical aspects of financial capacity in dementia: a commentary. *Am J Geriatr Psychiatry* 2013; 21: 382-390. DOI: 10.1016/j.jagp.2013.01.033.

13. Marson DC and Herbert K. Financial capacity. In: Cutler BL (ed) *Encyclopedia of psychology and the law*. California: Sage, 2008, pp.313-316.

14. Marson DC, Triebel KL and Knight AJ. Assessment of financial capacity: A neuropsychological perspective. In: Demakis GJ (ed) *Civil capacities in clinical neuropsychology*. New York: Oxford University, 2012, pp.39-68.

15. Widera E, Steenpass V, Marson DC, et al. Finances in the older patient with cognitive impairment: “He didn't want me to take over”. *J Am Med Assoc* 2011; 305: 698-706. DOI: 10.1001/jama.2011.164.

16. Connolly MT. Elder justice and dementia panel. In: *Advisory council on Alzheimer's Research, Care and Services Meetings* April 28 2015.

17. Lawton MP and Brody EM. Assessment of older people: self-maintaining and instrumental activities of daily living. *Gerontology* 1969; 9: 179-186.

18. Webber LS, Reeve RA, Kershaw MM, et al. Assessing financial competence. *Psychiatr Psychol Law* 2002; 9: 248-256. DOI: 10.1375/pplt.2002.9.2.248.

19. Carney T and Tait D. *The adult guardianship experiment: Tribunals and popular justice.* Sydney: Federation Press, 1997.

20. Kershaw MM and Webber LS. Dimensions of Financial Competence. *Psychiatr Psychol Law* 2004; 11: 338-349. DOI: 10.1375/pplt.2004.11.2.338.

21. Cranley Glass K and Silberfeld M. Determination of competence. In: Gauthier S (ed) *Clinical diagnosis and management of Alzheimer's disease*. London: Martin Dunitz, 1996, pp.331-342.

22. Stebnicki M. A conceptual framework for utilizing a functional assessment approach for determining mental capacity: A new look at informed consent in rehabilitiation *J Rehabil* 1997; 63: 32-36.

23. Willis SL. Assessing everyday competence in the cognitively challenged elderly In: Smyer M, Schaie KW and Kapp MB (eds) *Older adults decision making and the law*. New York: Springer, 1996, pp.87-127.

24. Kershaw MM and Webber LS. Assessment of financial competence. *Psychiatr Psychol Law* 2008; 15: 40-55. DOI: 10.1080/13218710701873965.

25. Appelbaum PS and Grisso T. Assessing patients' capacities to consent to treatment. *N Engl J Med* 1988; 319: 1635-1638. DOI: 10.1056/nejm198812223192504.

26. Moye J and Marson DC. Assessment of decision-making capacity in older adults: an emerging area of practice and research. *J Gerontol B Psychol Sci Soc Sci* 2007; 62: P3-p11. DOI: 10.1093/geronb/62.1.p3.

27. Marson DC and Briggs SD. Assessing competency in Alzheimer's disease: Treatment consent capacity and financial capacity. In: Gauthier S and Cummings JL (eds) *Alzheimer's disease and related disorders Annual: 2001*. London: Martin Dunitz, 2001.

28. Copeland JN. *Cortical thickness in amnestic mild cognitive impairment and its relationships with neuropsychological functioning and financial capacity*. The University of Alabama at Birmingham, Birmingham Alabama, 2013.

29. Lichtenberg PA, Stoltman J, Ficker LJ, et al. A person-centered approach to financial capacity assessment: preliminary development of a new rating scale. *Clin Gerontol* 2015; 38: 49-67. DOI: 10.1080/07317115.2014.970318.

30. Conrad KJ, Iris M, Ridings JW, et al. Self-report measure of financial exploitation of older adults. *Gerontologist* 2010; 50: 758-773. DOI: 10.1093/geront/gnq054.

31. Flint LA, Sudore RL and Widera E. Assessing Financial Capacity Impairment in Older Adults. *Generations* 2012; 36: 59-65.

32. Mast BT. *Whole person dementia assessment*. Baltimore, MD, US: Health Professions Press, 2011.

33. Lichtenberg PA, Ocepek-Welikson K, Ficker LJ, et al. Conceptual and empirical approaches to financial decision-making by older adults: results from a financial decision-making rating scale. *Clin Gerontol* 2018; 41: 42-65. DOI: 10.1080/07317115.2017.1367748.

34. Spreng RN, Karlawish J and Marson DC. Cognitive, social, and neural determinants of diminished decision-making and financial exploitation risk in aging and dementia: A review and new model. *J Elder Abuse Negl* 2016; 28: 320-344. DOI: 10.1080/08946566.2016.1237918.

35. Acierno R, Hernandez MA, Amstadter AB, et al. Prevalence and correlates of emotional, physical, sexual, and financial abuse and potential neglect in the United States: the National Elder Mistreatment Study. *Am J Public Health* 2010; 100: 292-297. DOI: 10.2105/ajph.2009.163089.

36. Jackson SL and Hafemeister TL. *Financial abuse of elderly people vs. other forms of elder abuse: Assessing their dynamics, risk factors, and society's response.* 2011. Final report presented to the National Institute of Justice.

37. James BD, Boyle PA and Bennett DA. Correlates of susceptibility to scams in older adults without dementia. *J Elder Abuse Negl* 2014; 26: 107-122. DOI: 10.1080/08946566.2013.821809.

38. Han SD, Boyle PA, James BD, et al. Mild cognitive impairment is associated with poorer decision-making in community-based older persons. *J Am Geriatr Soc* 2015; 63: 676-683. DOI: 10.1111/jgs.13346.

39. Marson DC, Martin R, Wadley V, et al. Clinical interview assessment of financial capacity in older adults with mild cognitive impairment and alzheimer's disease: financial capacity in older adults. *J Am Geriatr Soc* 2009; 57: 806-814. DOI: 10.1111/j.1532-5415.2009.02202.x.

40. Boyle PA, Yu L, Wilson RS, et al. Poor decision making Is a consequence of cognitive decline among older persons without Alzheimer’s disease or mild cognitive impairment. *PLoS One* 2012; 7: e43647. DOI: 10.1371/journal.pone.0043647.

41. National Academies of Sciences, Engineering, & Medicine. *Informing Social Security's Process for Financial Capability Determination*. Washington, DC: The National Academies Press, 2016. DOI: 10.17226/2192.

42. Engel LL, Bar Y, Beaton DE, et al. Identifying instruments to quantify financial management skills in adults with acquired cognitive impairments. *J Clin Exp Neuropsychol* 2016; 38: 76-95. DOI: 10.1080/13803395.2015.1087468.

43. Lillie RA, Kowalski K, Patry BN, et al. Everyday impact of traumatic brain injury. In: T.D. M and Grant I (eds) *Neuropsychology of everyday functioning*. New York, NY, US: The Guilford Press, 2010, pp.302-330.

44. Caboral-Stevens M and Medetsky M. The construct of financial capacity in older adults. *J Gerontol Nurs* 2014; 40: 30-37. DOI: 10.3928/00989134-20140325-02.

45. Hoskin KM, Jackson M and Crowe SF. Money management after acquired brain dysfunction: The validity of neuropsychological assessment. *Rehabil Psychol* 2005; 50: 355-365. DOI: 10.1037/0090-5550.50.4.355.

46. Knight AJ and Marson DC. The Emerging Neuroscience of Financial Capacity. *Generations* 2012; 36: 46-52.

47. Knoll MAZ and Houts CR. The Financial Knowledge Scale: An application of item response theory to the assessment of financial literacy. *J Consum Aff* 2012; 46: 381-410. DOI: https://doi.org/10.1111/j.1745-6606.2012.01241.x.

48. Gerstenecker A, Triebel K, Eakin A, et al. Exploring the factor structure of financial capacity in cognitively normal and impaired older adults. *Clin Gerontol* 2018; 41: 33-41. DOI: 10.1080/07317115.2017.1387211.

49. Marson DC and Zebley L. The other side of the retirement years: Cognitive decline, dementia, and loss of financial capacity. *J Retire Plann* 2001; 4: 30-39.

50. Engel LL, Beaton DE, Green RE, et al. Financial management activity process: qualitative inquiry of adults with acquired brain injury. *Can J Occup Ther* 2019; 86: 196-208. DOI: 10.1177/0008417419833839.

51. Edersheim J, Murray ED, Padmanabhan JL, et al. Protecting the Health and Finances of the Elderly With Early Cognitive Impairment. *J Am Acad Psychiatry Law* 2017; 45: 81-91.

52. Koller K, Woods L, Engel LL, et al. Loss of financial management independence after brain injury: survivors' experiences. *Am J Occup Ther* 2016; 70: 1-8. DOI: 10.5014/ajot.2016.020198.

53. Fenton L, Weissberger GH, Boyle PA, et al. Cognitive and neuroimaging correlates of financial exploitation vulnerability in older adults without dementia: Implications for early detection of Alzheimer's disease. *Neurosci Biobehav Rev* 2022; 140: 104773. DOI: 10.1016/j.neubiorev.2022.104773.

54. Wood S and Lichtenberg PA. Financial capacity and financial exploitation of older adults: Research findings, policy recommendations and clinical implications. *Clin Gerontol* 2017; 40: 3-13. DOI: 10.1080/07317115.2016.1203382.

55. Giebel C, Halpin K, Tottie J, et al. The digitalisation of finance management skills in dementia since the COVID-19 pandemic: A qualitative study. *Dementia (London)* 2023; 22: 783-806. DOI: 10.1177/14713012231159156.

56. Pérès K, Helmer C, Amieva H, et al. Natural history of decline in instrumental activities of daily living performance over the 10 years preceding the clinical diagnosis of dementia: a prospective population-based study. *J Am Geriatr Soc* 2008; 56: 37-44. DOI: https://doi.org/10.1111/j.1532-5415.2007.01499.x.

57. Giebel CM, Sutcliffe C and Challis D. Hierarchical Decline of the Initiative and Performance of Complex Activities of Daily Living in Dementia. *J Geriatr Psychiatry Neurol* 2017; 30: 96-103. DOI: 10.1177/0891988716686835.

58. Giebel CM, Flanagan E and Sutcliffe C. Predictors of finance management in dementia: managing bills and taxes matters. *Int Psychogeriatr* 2019; 31: 277-286. DOI: 10.1017/s1041610218000820.

59. Engel L, Arowolo I, Ewesesan R, et al. Contextual factors of financial capability and financial well-being for adults living with brain injury: a qualitative photovoice study. *Brain Inj* 2024; 38: 273-281. DOI: 10.1080/02699052.2024.2310210.

60. Kempson E, Collard S and Moore N. *Measuring financial capability: an exploratory study*. Report for the Financial Services Authority. Consumer Research 37, 2005. Personal Finance Research Centre, University of Bristol.

61. Kempson E and Poppe C. *Understanding financial well-being and capability - A revised model and comprehensive analysis.* 2018. Oslo, Norway: Oslo Metropolitan University.

62. MacKay S. *Understanding financial capability survey* 2011. Ottawa (ON): Taskforce on financial literacy.

63. Engel L, Ewesesan R, Arowolo I, et al. Financial capability and financial well-being challenges and vulnerabilities of adults living with acquired brain injury: A pilot survey. *Arch Rehabil Res Clin Transl* 2024; 6: 100324. DOI: 10.1016/j.arrct.2024.100324.
