# Supplementary material for: Conceptualisation of financial capability in adults with acquired cognitive impairment: A qualitative evidence synthesis
Source: Clin Rehabil. 2025 Jun 12;39(7):849–71. doi: 10.1177/02692155251347766 (PMC12198468; doi:10.1177/02692155251347766)
Supplement: sj-docx-3-cre-10.1177_02692155251347766 - Supplemental material for Conceptualisation of financial capability in adults with acquired cognitive impairment: A qualitative evidence synthesis [file sj-docx-3-cre-10.1177_02692155251347766.docx]

**Supplementary materials: Methodological quality assessment**

| Authors/year/Model | 1. Is the methodology identified and justified? (for model development) | 2. Was a theoretical lens or perspective used to guide the study? | 3. Is the theoretical framework described? | 4. Is the theoretical framework easily linked with the problem? | 5. If a conceptual framework is used, are the concepts adequately defined? | 6. Are the relationships among the concepts clearly identified? | Total "yes" (n) |
| --- | --- | --- | --- | --- | --- | --- | --- |
| Darzins P, Molloy DW, Strang D, et al. (2000) Who can decide? Property & finance | Somewhat | Somewhat | Yes | Yes | Yes | Yes | 4 |
| Marson DC, Sawrie SM, Snyder S, et al., (2000) Conceptual Model of Financial Capacity | Somewhat | Somewhat | Somewhat | Yes | Yes | No | 2 |
| Earnst KS, Wadley VG, Aldridge TM, et al. (2001) Conceptual Model of Financial Capacity (revision 1) | Somewhat | Somewhat | Somewhat | Yes | Yes | Somewhat | 2 |
| Griffith HR, Belue K, Sicola A, et al. (2003) Conceptual Model of Financial Capacity (revision 2) | Somewhat | Somewhat | Somewhat | Yes | Yes | Somewhat | 2 |
| Marson DC. (2016) Clinical model: Financial Capacity as Financial Skills relevant to Independence (revision 3) | Somewhat | Yes | Somewhat | Yes | Yes | Somewhat | 3 |
| Webber LS, Reeve RA, Kershaw MM, et al. (2002) Financial Competency Model | Somewhat | Somewhat | Somewhat | Yes | No | No | 1 |
| Kershaw MM & Webber LS. (2004) Financial Competency Model (revision 1) | Yes | Somewhat | Somewhat | Yes | Yes | Yes | 4 |
| Kershaw MM & Webber LS. (2008) Financial Competency Model (evaluated) | Yes | Somewhat | Somewhat | Yes | Yes | Yes | 4 |
| Moye J & Marson DC. (2007) Working model of financial capacity | No | Somewhat | Somewhat | Yes | Yes | No | 2 |
| Marson DC (2016) Cognitive Psychological Model: Financial Capacity as Types of Financial Knowledge (revision 1) | No | Somewhat | No | Yes | Yes | Yes | 3 |
| Copeland JN. (2013) Conceptual model of declining financial capacity in amnestic mild cognitive impairment (MCI) and Alzheimer's disease | Yes | Yes | Yes | Yes | Yes | Yes | 6 |
| Lichtenberg PA, Stoltman J, Ficker LJ, et al. (2015) Financial decisional abilities model | Yes | Yes | Yes | Yes | Yes | Yes | 6 |
| Lichtenberg et al., (2018) Financial Decisional Capacity Model (referred to) | Yes | Yes | Yes | Yes | Yes | Yes | 6 |
| Spreng RN, Karlawish J & Marson DC (2016) Social cognitive neuroscience model for assessing financial exploitation risk | Yes | Yes | Yes | Yes | Yes | Yes | 6 |
| National Academies of Sciences, Engineering and Medicine (2016) Institute of medicine conceptual model of financial capability | Yes | Yes | Yes | Yes | Yes | Yes | 6 |
| Gerstenecker A, Triebel K, Eakin, A, et al. (2018) Four factor structure of financial capacity | Yes | Yes | Yes | Yes | Yes | Somewhat | 5 |
| Engel LL, Bar Y, Beaton DE, et al. (2016) Concept of financial management | No | Yes | Yes | Yes | n/a | n/a | 3 |
| Engel LL, Beaton DE, Green RE, et al. (2019) The financial management activity process (FMAP) | Yes | Yes | Yes | Yes | Yes | Yes | 6 |
| Fenton L, Weissberger GH, Boyle P A, et al. (2022) Financial exploitation vulnerability manifests as an early behavioural sign of underlying Alzheimer's disease related neuropathology | Yes | Somewhat | Somewhat | Yes | Somewhat | Yes | 3 |
| Giebel C, Halpin K, Tottie J, et al. (2023) The digitisation of finance management skills in dementia since the COVID-19 Pandemic: A qualitative study | Yes | Yes | Yes | Yes | Yes | Somewhat | 5 |
| Engel L, Arowolo I, Ewesesan R, et al. (2024) Contextual factors of financial capability and financial well-being for adults living with acquired brain injury: a qualitative photovoice study. | Yes | Yes | Yes | Yes | Yes | Somewhat | 5 |
| Engel L. Ewesesan R, Arowolo I, et al. (2024) Financial capability and financial well-being challenges and vulnerabilities of adults living with acquired brain injury. | Yes | Yes | Yes | Yes | Yes | Somewhat | 5 |
| Total (yes) | 13 | 12 | 12 | 21 | 19 | 11 |  |

Scoring: Yes, No or Somewhat (some information provided, but not enough to say yes).

3 or fewer yes ratings: moderate

4 or more yes ratings: strong
