# Supplementary material for: Conceptualisation of financial capability in adults with acquired cognitive impairment: A qualitative evidence synthesis
Source: Clin Rehabil. 2025 Jun 12;39(7):849–71. doi: 10.1177/02692155251347766 (PMC12198468; doi:10.1177/02692155251347766)
Supplement: sj-docx-4-cre-10.1177_02692155251347766 - Supplemental material for Conceptualisation of financial capability in adults with acquired cognitive impairment: A qualitative evidence synthesis [file sj-docx-4-cre-10.1177_02692155251347766.docx]

**Supplementary Materials: Coding matrix**

| Themes | Multi-dimensionality of financial capability | | | | Financial decision-making ability & exploitation risk for legal capacity/competence | | Neuropathological cause of declining financial capability |
| --- | --- | --- | --- | --- | --- | --- | --- |
| Subthemes | Multi-dimensionality of financial capability tasks | Multidimensionality of financial capability knowledge, skills and abilities | Real-world processes and performance | Extent of individualisation | Legal Capacity | Financial exploitation risk |  |
| Codes (Models as numbered in Table 2) | Financial capability as multi-domain/ dimensional in respect to financial tasks  (Models: 2, 3, 4, 9, 10) | Discusses contribution of knowledge, skills and/or abilities to financial capability.  (Models: 1, 2, 3, 4, 5, 6, 7, 8, 9, 10, 11, 12, 13, 14, 15) | Acknowledges that financial knowledge, judgement and skills not always indicative of real-world performance (Models: 8, 11, 13). | Considers a person's individual context/contextual factors  (Models: 1, 3, 4, 6, 7, 8, 9, 11, 13, 14, 15) | Considers legal criteria of capacity: understanding, appreciation, reasoning, expressing a choice (Applebaum/Grisso) (Models: 1, 3, 6, 8) | Focus of model on financial decision-making abilities and/or financial exploitation risk (Models: 6, 7, 12) | Considers underlying neuropathological cause of decline in financial capability  (Models: 5, 7, 12). |
|  | Considers skills, abilities and task performance within domains dimensions (Models: 2, 3, 4, 10) | Makes distinction between cognitive/judgement skills & functional/performance skills.  (Models: 1, 2, 3, 4, 6, 8, 10, 11, 13) | Considers how financial tasks are carried out including process and actions in a real-world environment  (Model: 1, 8, 11, 13, 14) | Gathers information about a person's individual context  (Models: 1, 3, 4, 6, 11) | Considers overall financial capacity vs. capacity for specific decision  (Models: 1, 2, 3, 4, 6, 8, 10). | Considers potential danger/risk related to loss of financial skills prior to dementia diagnosis  (Models: 7, 12, 13) | Neuropathological changes in brain underlie specific neurocognitive dysfunction, which drives functional decline in mild cognitive impairment and Alzheimer’s Disease  (Model: 5, 7) |
|  | Explicitly recognises as an IADL  (Models: 2, 4, 5, 13) | General knowledge of assets, debts and everyday use of money (Models: 1, 2, 3, 4, 10) | Considers a person's knowledge of their own personal financial situation  (Models: 1, 4, 11, 13) | Considers a person's available financial resources /employment/economic context  (Models: 14, 15) | Makes distinction between capacity/competence being a legal framework, and financial management skills being a component of this  (Models: 9, 11) | Self or carer perceived increase in risk for financial exploitation vulnerability due to cognitive impairment (Models: 13, 14, 15) | Structural and functional brain changes in social capacity interact with cognitive capacity and can lead to financial exploitation risk  (Model: 7) |
|  | Recognises as an ‘occupation’ important for independent living and general health/ financial well-being/quality of life.  (Models: 9, 11, 14, 15 | Conceptual overview of components of financial capability  (Models: 4, 8, 9, 10) | Consumer identifies financial management activity that they need or want to do  (Model: 11) | Explicitly person-centred  (Models: 6, 7, 11) |  |  | Financial exploitation vulnerability is an early behavioural sign of underlying Alzheimer’s disease neuropathology from Beta-amyloid in default mode network.  (Model: 12) |
|  |  |  | Considers complexity of financial processes and information being a potential barrier  (Models: 13, 14, 15). | Focus on independence (Models: 1, 2, 4, 5, 10, 11) |  |  |  |
|  |  |  | Considers availability or lack of support provided by external statutory bodies or banks etc.  (Models: 8, 11, 13, 14, 15) | Promotion of own financial well-being  (Models: 2, 4, 10) |  |  |  |
|  |  |  | Acknowledges changing technological financial landscape  (Models: 11, 13, 14) | Includes education for person  (Model: 1) |  |  |  |
|  |  |  | Considers high/low technology for strategies (Models: 11, 13, 14) | Considers personalised strategies  (Model: 11, 13, 14, 15) |  |  |  |
|  |  |  | Considers benefits and disadvantages of digitisation of finances (Models: 11, 13, 14) | Considers perspectives of significant others  (Models: 1, 8, 11, 13, 15) |  |  |  |
|  |  |  | Bias and stigma from 'invisible disability' leading to lack of support or recognition of need for further information, time or support to complete tasks  (Models: 13, 14) | Considers person directing management of money/receiving support rather than total independence  (Models: 8, 11, 13, 14, 15) |  |  |  |
|  |  |  | Social benefits of in-person financial management tasks (e.g. shopping)  (Model: 13) | Considers impact on carers  (Models: 13) |  |  |  |
|  |  |  | Layout and design of financial institutions as a barrier or facilitator  (Model: 11, 14) | Considers persons acceptance of a carer managing their finances  (Models: 13) |  |  |  |
|  |  |  | Model fluid and dynamic and aims to guide rehabilitation  (Model: 11) |  |  |  |  |
|  |  |  | Considers financial capability in relation to International Classification of Functioning (ICF)  (Models: 8, 9) |  |  |  |  |
