# Supplementary material for: Conceptualisation of financial capability in adults with acquired cognitive impairment: A qualitative evidence synthesis
Source: Clin Rehabil. 2025 Jun 12;39(7):849–71. doi: 10.1177/02692155251347766 (PMC12198468; doi:10.1177/02692155251347766)
Supplement: sj-pdf-5-cre-10.1177_02692155251347766 - Supplemental material for Conceptualisation of financial capability in adults with acquired cognitive impairment: A qualitative evidence synthesis [file sj-pdf-5-cre-10.1177_02692155251347766.pdf]

Supplementary Materials: GRADE-CERQual Assessment

Conceptualisation of financial capability in adults with acquired cognitive impairment: A qualitative evidence synthesis

Summary of Qualitative Findings Table

SEE MORE INFORMATION +

Review question

This review aims to establish the key concepts, working definitions and theoretical models or conceptual frameworks in relation to financial capability in adults with acquired cognitive impairment due to acquired brain injury or neurological disease.

Has the review been published?

No

Authors of the review

- Sarah Swan
- Freyr Patterson
- Jennifer Fleming

Corresponding author

Sarah Swan
s.swan1@uq.edu.au

#	Summarised review finding	GRADE-CERQual assessment of confidence	Explanation of GRADE-CERQual assessment	References
1	Theme 1: Multi-dimensionality of financial capability. This theme focused on how financial capability can be considered a multi-dimensional construct, with four sub-themes including multi-dimensionality of financial capability tasks; multi-dimensionality of financial knowledge, skills and abilities; real-world processes and performance; and the extent of individualisation. Definitions that reflected this theme related to financial capacity, financial competence, financial management, financial performance, financial capability and financial well-being.	High confidence	No/Very minor concerns regarding methodological limitations, No/Very minor concerns regarding coherence, No/Very minor concerns regarding adequacy, and No/Very minor concerns regarding relevance	Copeland 2013; Molloy et al. 2000; Earnst et al. 2001; Engel et al. 2019; Kershaw & Webber 2004; Kershaw & Webber 2008; Lichtenberg et al. 2018; Lichtenberg et al. 2015; Spreng et al. 2016; Webber et al. 2002; Engel et al. 2024; Engel et al. 2024; Engel et al. 2016; Fenton et al. 2022; Gerstenecker et al. 2018; Giebel et al. 2023; Griffith et al. 2003; Marson 2016; Marson et al. 2000; Moye & Marson 2007; Appelbaum et al. 2016;
2	Theme 2: Financial decision-making ability and exploitation risk for legal capacity. This theme focused on the ability to make financial decisions in respect to a person's legal capacity, alongside the risk for financial exploitation when a person's ability to do so is in decline. This theme included two sub-themes of legal capacity and financial exploitation risk. Terminology focused on competence, capacity, financial judgement, financial decision-making, and financial exploitation.	High confidence	No/Very minor concerns regarding methodological limitations, No/Very minor concerns regarding coherence, No/Very minor concerns regarding adequacy, and No/Very minor concerns regarding relevance	Molloy et al. 2000; Earnst et al. 2001; Engel et al. 2019; Kershaw & Webber 2004; Kershaw & Webber 2008; Lichtenberg et al. 2018; Lichtenberg et al. 2015; Spreng et al. 2016; Webber et al. 2002; Engel et al. 2024; Engel et al. 2024; Engel et al. 2016; Fenton et al. 2022; Gerstenecker et al. 2018; Giebel et al. 2023; Griffith et al. 2003; Marson 2016; Marson et al. 2000; Moye & Marson 2007; Appelbaum et al. 2016;

#	Summarised review finding	GRADE-CERQual assessment of confidence	Explanation of GRADE-CERQual assessment	References
3	Theme 3: Neuropathological cause of declining financial capability. This theme focused on the underlying neuropathological cause of declining financial capability (related to dementia or mild cognitive impairment). Hypotheses related to underlying cause varied.	Moderate confidence	No/Very minor concerns regarding methodological limitations, No/Very minor concerns regarding coherence, Minor concerns regarding adequacy, and Moderate concerns regarding relevance	Copeland 2013; Spreng et al. 2016; Fenton et al. 2022;

Evidence Profile Table

#	Summarised review finding	Methodological limitations	Coherence	Adequacy	Relevance	GRADE-CERQual assessment of confidence	References
1	Theme 1: Multi-dimensionality of financial capability. This theme focused on how financial capability can be considered a multi-dimensional construct, with four sub-themes including multi-dimensionality of financial capability tasks; multi-dimensionality of financial knowledge, skills and abilities; real-world processes and performance; and the extent of individualisation. Definitions that reflected this theme related to financial capacity, financial competence, financial management, financial performance, financial capability and financial well-being.	No/Very minor concerns<br><br>**Explanation:** All papers included in the study reflected on the multi-dimensionality of financial capability. A strong quality rating was given to 13 of these papers, with 8 papers rated as moderate quality. Most papers had adequate definitions and descriptions of model and framework concepts. Some of papers rated as moderate quality lacked clear statements regarding the perspective or theoretical lens used to guide the study, did not identify or justify the methodology for model development, or lacked description of the interaction between concepts.	No/Very minor concerns<br><br>**Explanation:** Concepts and models were not consistent between papers, however reflecting on the the multi-dimensionality of financial capability was consistent, particularly in relation to knowledge, skills and abilities. The multi-dimensionality of financial capability tasks, real-world processes and performance, and the extent of individualisation was also discussed in relation to multiple models.	No/Very minor concerns<br><br>**Explanation:** No concerns exist about the adequacy or richness of the data, exemplified by all included papers reflecting this theme in some manner. Nil conflicting views noted.	No/Very minor concerns<br><br>**Explanation:** All studies relevant to review finding.	High confidence<br><br>**Explanation:** No/Very minor concerns regarding methodological limitations, No/Very minor concerns regarding coherence, No/Very minor concerns regarding adequacy, and No/Very minor concerns regarding relevance	Appelbaum et al. 2016; Copeland 2013; Earnst et al. 2001; Engel et al. 2016; Engel et al. 2019; Engel et al. 2024; Engel et al. 2024; Fenton et al. 2022; Gerstenecker et al. 2018; Giebel et al. 2023; Griffith et al. 2003; Kershaw & Webber 2004; Kershaw & Webber 2008; Lichtenberg et al. 2015; Lichtenberg et al. 2018; Marson 2016; Marson et al. 2000; Molloy et al. 2000; Moye & Marson 2007; Spreng et al. 2016; Webber et al. 2002;

#	Summarised review finding	Methodological limitations	Coherence	Adequacy	Relevance	GRADE-CERQual assessment of confidence	References
2	Theme 2: Financial decision-making ability and exploitation risk for legal capacity. This theme focused on the ability to make financial decisions in respect to a person's legal capacity, alongside the risk for financial exploitation when a person's ability to do so is in decline. This theme included two sub-themes of legal capacity and financial exploitation risk. Terminology focused on competence, capacity, financial judgement, financial decision-making, and financial exploitation.	No/Very minor concerns<br><br>**Explanation:** This theme was reflected in all but one of the included papers. A strong quality rating was given to 12 of these papers, with 8 papers rated as moderate quality. Most papers had adequate definitions and descriptions of model and framework concepts. Some of papers rated as moderate quality lacked clear statements regarding the perspective or theoretical lens used to guide the study, did not identify or justify the methodology for model development, or lacked description of the interaction between concepts.	No/Very minor concerns<br><br>**Explanation:** This theme was represented in 20 of 21 included papers. Whilst there may be inconsistency between some papers regarding the definition or description of components related to these concepts, the overall data is consistent with this theme.	No/Very minor concerns<br><br>**Explanation:** No concerns exist about the adequacy or richness of the data, exemplified by most included papers reflecting this theme in some manner.	No/Very minor concerns<br><br>**Explanation:** All studies relevant to review finding.	High confidence<br><br>**Explanation:** No/Very minor concerns regarding methodological limitations, No/Very minor concerns regarding coherence, No/Very minor concerns regarding adequacy, and No/Very minor concerns regarding relevance	Appelbaum et al. 2016; Earnst et al. 2001; Engel et al. 2016; Engel et al. 2019; Engel et al. 2024; Engel et al. 2024; Fenton et al. 2022; Gerstenecker et al. 2018; Giebel et al. 2023; Griffith et al. 2003; Kershaw & Webber 2004; Kershaw & Webber 2008; Lichtenberg et al. 2015; Lichtenberg et al. 2018; Marson 2016; Marson et al. 2000; Molloy et al. 2000; Moye & Marson 2007; Spreng et al. 2016; Webber et al. 2002;

#	Summarised review finding	Methodological limitations	Coherence	Adequacy	Relevance	GRADE-CERQual assessment of confidence	References
3	Theme 3: Neuropathological cause of declining financial capability. This theme focused on the underlying neuropathological cause of declining financial capability (related to dementia or mild cognitive impairment). Hypotheses related to underlying cause varied.	No/Very minor concerns<br><br>**Explanation:** Two out of three studies contributing to this finding were rated as strong in methodological quality. The third was moderate in quality, mostly due to lack of description of guiding theoretical framework.	No/Very minor concerns<br><br>**Explanation:** The theme reflects the fit between the extracted data, and does not include comment on consistency of findings.	Minor concerns<br><br>**Explanation:** Minor concerns regarding adequacy because only 3 papers were found related to this theme.	Moderate concerns<br><br>**Explanation:** Moderate concerns regarding relevance because included papers were only relevant to dementia and mild cognitive impairment populations; whereas the review covered broader populations with acquired cognitive impairment.	Moderate confidence<br><br>**Explanation:** No/Very minor concerns regarding methodological limitations, No/Very minor concerns regarding coherence, Minor concerns regarding adequacy, and Moderate concerns regarding relevance	Copeland 2013; Fenton et al. 2022; Spreng et al. 2016;
